# Molecular Plasticity under Ocean Warming: Proteomics and Fitness Data Provides Clues for a Better Understanding of the Thermal Tolerance in Fish

**DOI:** 10.3389/fphys.2017.00825

**Published:** 2017-10-23

**Authors:** Diana Madeira, José E. Araújo, Rui Vitorino, Pedro M. Costa, José L. Capelo, Catarina Vinagre, Mário S. Diniz

**Affiliations:** ^1^UCIBIO-REQUIMTE, Department of Chemistry, Faculty of Science and Technology, Universidade Nova de Lisboa, Lisbon, Portugal; ^2^Centre for Environmental and Marine Studies, University of Aveiro, Aveiro, Portugal; ^3^Department of Medical Sciences, Institute of Biomedicine, University of Aveiro, Aveiro, Portugal; ^4^Department of Physiology and Cardiothoracic Surgery, Faculty of Medicine, University of Porto, Porto, Portugal; ^5^MARE - Marine and Environmental Sciences Centre, Faculty of Sciences and Technology, Universidade Nova de Lisboa, Lisbon, Portugal; ^6^MARE - Marine and Environmental Sciences Centre, Faculty of Sciences, University of Lisbon, Lisbon, Portugal

**Keywords:** acclimation, global change, fish, phenotypic plasticity, proteome, temperature

## Abstract

Ocean warming is known to alter the performance and fitness of marine organisms albeit the proteome underpinnings of species thermal tolerance are still largely unknown. In this 1-month experiment we assessed the vulnerability of the gilt-head sea bream *Sparus aurata*, taken here as a biological model for some key fisheries species, to ocean warming (control 18°C, nursery ground temperature 24°C and heat wave 30°C). Survival was impaired after 28 days, mainly at 30°C although fishes' condition was unaltered. Muscle proteome modulation was assessed at 14 and 21 days, showing that protein expression profiles were similar between fish exposed to 18 and 24°C, differing from fish exposed to 30°C. Fish subjected to 24°C showed an enhanced glycolytic potential and decreased glycogenolysis mainly at 14 days of exposure. Fish subjected to 30°C also showed enhanced glycolytic potential and up-regulated proteins related to gene expression, cellular stress response (CSR), and homeostasis (mostly cytoskeletal dynamics, acid-base balance, chaperoning). However, inflammatory processes were elicited at 21 days along with a down-regulation of the tricarboxylic acid cycle. Thus, juvenile fish seem able to acclimate to 24°C but possibly not to 30°C, which is the predicted temperature for estuaries during heat waves by the year 2100. This may be related with increasing constraints on organism physiology associated with metabolic scope available for performance and fitness at higher temperatures. Consequently, recruitment of commercial sea breams may be in jeopardy, highlighting the need for improved management plans for fish stocks.

## Introduction

Climate change has been associated with ecological variation, including alterations in biodiversity, abundance and distribution of species, and phenology (Parmesan and Yohe, 2003; Poloczanska et al., 2013). Within Europe, the Iberian Peninsula and Mediterranean area are regions with an elevated rate of warming, with a predicted increase between 4 and 7°C in air temperature by 2100 (Santos and Miranda, 2006; Christensen et al., 2007). Under such scenario, Portuguese coastal waters and the Mediterranean Sea will be 2–3°C and 3–4°C warmer, respectively (Miranda et al., 2002; Fischer and Schär, 2010). The impacts of such temperature changes, coupled with the effects of other anthropogenic activities may exert strong influences on ecosystem services, affecting economic activities such as fisheries and aquaculture. Climate change is predicted to affect such activities not only through effects of temperature on species physiology but also through changes on fish farming practices (Brander, 2007; Cochrane et al., 2009; Doubleday et al., 2013). Therefore, it is imperative to evaluate and understand biological responses of commercial fish species to the rise in temperature. This will allow researchers to identify the vulnerability of key species, and stakeholders to develop appropriate mitigation plans to reduce costs to society and environment (Poloczanska et al., 2013).

At a physiological level, temperature affects individual's metabolic rate, aerobic metabolism, performance, growth, reproduction, and survival with potential effects on population sustainability (Kordas et al., 2011; Dowd et al., 2015). Moreover, changes at the individual level are dependent upon the capacity to modulate gene expression under environmental variation (see Logan and Somero, 2011). Changes in cellular stress proteins (such as heat shock proteins and anti-oxidant enzymes) have been detected in marine organisms exposed to acute and chronic heat stress (e.g., Buckley and Hofmann, 2002; Tomanek, 2010; Madeira et al., 2013, 2014). Nevertheless, these studies have only focused on a few protein biomarkers. Additionally, research incorporating high throughput tools is highly skewed toward transcriptomics and therefore proteomics approaches are still scarce in marine biology (see Tomanek, 2014; Logan and Buckley, 2015 for reviews). Proteomics examines the ultimate product of gene expression, i.e., proteins, which are the functional units mediating stressor-biological system interactions and its subsequent responses (e.g., López, 2007). Thus, it can revolutionize our understanding of acclimation and adaptation processes and may be the basis of an integrative, systems biology approach (Karr, 2008), enabling a fast assessment of stress response networks affected by environmental cues. Currently, several pathways are known to be regulated by acute and chronic exposure to elevated temperature. When organisms are exposed to heat, hypoxaemia seems to set the first level of thermal limitation (Pörtner, 2010). Gradually, temperature will lead to other pervasive effects such as oxidative stress and protein damage. Accordingly, organisms usually modulate protein levels when temperature rises a few degrees above their acclimation temperature, in order to induce a stress response to protect cellular components from damage and/or meet potentially higher energetic demands caused by elevated metabolic rates. According to several “omics” studies, modulated proteins play roles in chaperoning activity, energetic metabolism, oxidative stress metabolism, immune response, cytoskeletal dynamics, transcriptional regulation, intracellular transport, protein synthesis and turnover, and signal transduction (see López et al., 2002; Gardeström et al., 2007; Stillman and Tagmount, 2009; Fields et al., 2012; Tomanek, 2014). By examining the full range of protein changes, the cells' phenotype can be assessed and directly related to the health and fitness of the organism and adaptation mechanisms (Dupont et al., 2007; Dalziel and Schulte, 2012; Diz et al., 2012). Consequently, predictions of cascading effects on populations can be improved, up-grading biogeographic forecasting, risk assessment, and prediction of potential costs to society.

*Sparus aurata* is an euryhaline and eurythermic species. Thus, it is widely distributed in the eastern Atlantic and is known from southern Ireland to Mauritania, including the Canary Islands, the Mediterranean Sea and the western and southern Black Sea (Froese and Pauly, 2006). In general, minimum and maximum survival temperatures are in the range of 5–34°C for adult sea breams. However, early life-stages (eggs, larvae and juveniles) have more limited temperature tolerance during ontogenesis. Moreover, temperature tolerance is highly dependent on acclimation. Accordingly, rapid and significant changes of temperature near to the thermal limits are likely to lead to poor welfare (European Food Safety Authority, 2008).

The aim of this study was to assess the vulnerability and acclimation capacity of the sea bream *S. aurata (L*., 1758), taken here as a proxy for some commercially important demersal fish species, to ocean warming and extreme events by (i) unraveling proteome changes in the muscle of juvenile fish exposed to warming, (ii) relating proteome plasticity to fitness measures (mortality and condition index) and (iii) inferring on the possible ecological consequences of warming for sea bream populations. Muscle tissue was chosen as target organ for several reasons, including (1) muscle proteome has been systematically characterized in *S. aurata* and is therefore well known in this species (Addis et al., 2010; Piovesana et al., 2016), (2) the commercial interest of this tissue, (3) muscle activity accounts for a great part of the organism's resting energy expenditure (Zurlo et al., 1990), and (4) muscular activity is crucial to dictate organismal functions such as locomotion and thus the capacity to escape unfavorable conditions and forage, determining the performance of the organism. We hypothesize that fitness outcomes depend on the capacity of fish to induce pro-survival pathways in extreme conditions (by up-regulating proteins with cytoprotective functions) and adjust energetic metabolism to meet the higher metabolic rates induced by warm water, determining the fate of sea bream populations in the face of climate change.

## Materials and methods

### Ethical statement

This study was carried out in accordance with the recommendations of EU legislation for animal experimentation (Directive 2010/63/EU). The protocol was approved by the *Direcção Geral de Alimentação e Veterinária*.

### Assessment of thermal environments

Temperature data were obtained from (1) studies throughout Portuguese coastal waters and estuaries (Costa, 1990; Coutinho, 2003; Azevedo et al., 2006; Cabral et al., 2007; Madeira et al., 2012); (2) sea temperature database (satellite data available from http://seatemperature.info/portugal-water-temperature.html) which has monthly sea surface temperatures for the main coastal cities of Portugal (data from the past five years −2011 to 2015); and (3) the Marine and Environmental Sciences Centre (MARE) database (data from several estuaries including Tagus' temperatures obtained from measurements carried out with thermometers and YSI loggers from 1978 to 2006).

### Housing and husbandry of fish

Juvenile *S. aurata* (*n* = 36, mean ± *sd* total length of 8.93 ± 1.16 cm and 12.76 ± 4.60 g weight), 5–6 months old, were obtained from a fish farm (MARESA, Spain) and transported in September to the laboratory in 100 L opaque polyvinyl tanks with constant aeration and stable temperature conditions (18 ± 0.5°C).

The first parental fish of the hatchery were wild fish caught in the nearby coastal area mixed with adults obtained from other aquaculture facilities.

Fish were placed in a re-circulating system with 500 L white polyvinyl tanks (120 × 45 × 95 cm; *n* = 36 individuals in one tank). The tank was filled with clean and aerated sea water (95–100% air saturation), with a constant temperature of 18 ± 0.5°C, salinity 35‰ and pH 8 ± 0.01 (same conditions of the fish farm). The fish were housed for 1 week, before the beginning of the experiments, and their welfare was assessed. During housing and experimental trial juveniles were fed with commercial feed pellets once a day (Aquasoja, Portugal) mixed with cyanobacterium *Spirulina* sp. (Tropical®, Poland) (because these fish are accessorily herbivorous). The photoperiod regime was 15 h light −09 h dark. Temperature was monitored daily using a digital thermometer and salinity (≈35‰), pH (8 ± 0.01), ammonia (≈0 mg.L^−1^) and nitrites (≤0.3 mg.L^−1^), were monitored twice a week using a hand-held refractometer (Atago, Japan), a digital pH probe (model HI9025, Hanna Instruments, USA), and Tetra test Kits (Tetra Ammonia Test Kit and Tetra Nitrites Test Kit), respectively.

### Experimental setup

Following the acclimation period, fish were randomly transferred to experimental tanks (Figure S1). This system consisted of a re-circulating structure (total volume of 2,000 L) fitted with a skimmer plus biological, mechanical and UV filter. Six 70 L white polyvinyl tanks (35 × 35 × 55 cm) with *n* = 6 individuals.tank^−1^ composed the experimental system. Inflow of clean water in each tank was 300 mL.min^−1^. All the tanks were filled with clean and aerated sea water (95–100% O_2_), with a constant temperature of 18 ± 0.5°C, salinity 35‰ and pH 8 ± 0.01. All tanks were also provided with a filter (ELITE Underwater Mini-Filter Hagen, 220 L.h^−1^) to remove debris from feeding and excretion. Before the trial started, the temperature was gradually increased (6°C per 24 h—similar to what can happen in estuaries) until the experimental temperatures were reached (control 18 ± 0.5°C; experimental temperatures 24 ± 0.5 and 30 ± 0.5°C; *n* = 2 tanks for each temperature). Temperatures were maintained using thermostats (TetraTec® HT 100, 100–150 L, Tetra Werke, GmbH, Melle, Germany). The sampling scheme (Figure S1) consisted of euthanizing fish trough cervical transection at days 14, 21 and 28 (at 18, 24, and 30°C) for the collection of skeletal muscle. At each time point, four fish were randomly sampled at each temperature (two from each tank) and abdominal muscle was removed. Although the sample size may be considered small, it is similar to others used in several omics studies (e.g., Stillman and Tagmount, 2009; Logan and Somero, 2011; Jayasundara et al., 2015). Total length and weight of all individuals were determined upon sampling.

### Fitness assessment

Mortality rates were calculated in each tank at the end of the experiment (*n* = 6 fish·tank^−1^ so total *n* = 12 individuals for each temperature). Fulton's K condition index was calculated at 21 days of exposure (*n* = 4 individuals for each temperature) using the formula:

(1) $$\begin{array}{r} \text{K }=\;100{\text{M}}_{\text{t}}/{\text{L}}_{\text{t}}^{3} \end{array}$$

Where M_t_ is the total wet mass (mg) and L_t_ is the total length (mm) (Ricker, 1975). No analyses were carried out at 28 days of exposure due to mortality (reduced sample size).

#### Statistical analysis of fitness measures

Data was tested for normality (Shapiro–Wilk's test) and homoscedasticity (Levene's test). As Fulton's K data did not meet both assumptions, a non-parametric Kruskal–Wallis test was applied. Mortality data was analyzed through Student's *t*-test (for independent samples, by groups), applying the Bonferroni correction. All analyses were carried out in Statistica v10 (StatSoft Inc., USA), using a significance level of 0.05.

### Protein extraction

Muscle samples (~200 mg) were homogenized in 1 mL of phosphate buffered saline (pH 7.4) to extract proteins (the most soluble are cytosolic proteins), using a Tissue Master 125 homogenizer (Omni International, Kennesaw, USA) on ice-cold conditions. The crude homogenates were then centrifuged for 15 min at 10,000 × g and the supernatants were frozen immediately (−80°C) until further analyses.

### Proteomic analysis

This analysis followed the traditional gel-based approach coupled to mass spectrometry, as described in Diz et al. (2012), Tomanek (2011), You and Wang (2006) with minor changes (following Madeira et al., 2016). Due to mortality rates, proteomic analyses were only carried out for the first two sampling points: 14 and 21 days of exposure.

#### Sample preparation

The homogenized and centrifuged samples were precipitated through the DOC/TCA (Na-deoxycholate/trichloroacetic acid) method adapted from Peterson (1977) with minor changes (Madeira et al., 2016). Briefly, for each 100 μL of sample, 1 μL of 2% (w/v) DOC was added and samples were incubated 30 min on ice. Then, 18 μL of 100% (w/v) TCA was added to the mixture and microtubes were incubated overnight on ice. Following, samples were centrifuged at 14,000 × g for 20 min at 4°C. Supernatant was removed and pellets were washed with 200 μL of ice cold acetone, followed by another centrifugation (14,000 × g for 20 min at 4°C). This washing step was performed twice. Subsequently, pellets were re-suspended in rehydration buffer (7 M urea, 2 M thiourea, 2% w/v CHAPS—cholamidopropyl-dimethylammonio-propanesulfonic acid, 0.2% v/v IPG buffer, 0.002% bromophenol blue, 50 mM DTT—dithioerythritol). Protein content was determined through the method of Bradford (1976), using a microplate spectrophotometer (model LT-4000, Labtech, United Kingdom).

#### Two dimensional gel electrophoresis (2-DE)

Samples containing 200 μg of muscle protein were loaded onto IPG strips (pH 3–10, 7 cm, Bio-Rad) for separation according to their isoelectric point (pI). IPG strips had been previously rehydrated overnight with 7 M urea, 2 M thiourea, 0.5% w/v CHAPS, 0.2% v/v IPG buffer, 0.002% bromophenol blue, 10 mM DTT. Isoelectric focusing was carried out in a Protean IEF Cell (Bio-Rad), according to the manufacturer's instructions for 7 cm strips: 250 V for 20 min (linear mode), 4,000 V for 2 h (linear mode) and 4,000 V for 10,000 V-h (rapid mode). Strips were immediately incubated in equilibration buffer I for protein reduction (6 M urea, 75 mM Tris-HCl, 20% v/v glycerol, 2% w/v SDS—sodium dodecyl sulfate, 0.002% bromophenol blue, 2% w/v DTT) for 15 min with continuous shaking, and then equilibration buffer II for protein alkylation (6 M urea, 75 mM Tris-HCl, 20% v/v glycerol, 2% w/v SDS, 0.002% bromophenol blue, 2,5% w/v IAA—iodoacetamide) for 15 min with continuous shaking. Afterwards, IPG strips were placed on top of 12.5% polyacrylamide gels and were covered with an agarose sealing solution (0.5% w/v agarose and 0.002% bromophenol blue in running buffer −25 mM Tris base, 192 mM glycine, 0.1% SDS). Gels were run in Mini-Protean® 3 Cell (Bio-Rad) at 200 V for 45 min and were then stained for 48 h with a solution of colloidal Coomassie Blue G-250 (0.12% w/v Coomassie G-250, 10% w/v ammonium sulfate, 10% w/v orthophosphoric acid, 20% methanol). Following, gels were de-stained with milli-Q grade water in several washes. Two replicate gels were carried out for each individual and included in the gel image analysis to ensure gel reproducibility.

#### Gel image analysis

Gel imaging was carried out with the PropicII-robot (Genomic Solutions™, Cambridgeshire, UK) and digitalized images of the gels were analyzed with Progenesis SameSpots software (version 4.0, Non-Linear Dynamics, Totallab, UK). Two image analyses were carried out, one for each time point, comparing 18, 24 and 30°C at 14 days of exposure (T14) and then at 21 days of exposure (T21). A master gel was automatically defined in each analysis by the software and match vectors were also automatically created to align the gels (match the spots within all the gels). Protein spot volumes were normalized against total spot volume of all proteins in a gel image. In each analysis, spot volumes were compared via a one-way analysis of variance to detect proteins that were differentially expressed based on a statistical significance of 0.05. Following, Tukey's *post-hocs* (Table S1) were carried out in StatPages.net (http://statpages.org/anova1sm.html) with the significance level or adjusted *p*-values for false discovery rates (*q*-values) set at 0.05. These analyses were performed using mean ± *sd* of log normalized volumes retrieved from SameSpots.

#### Protein digestion

The spots of interest were manually excised from gels and transferred to 0.5 mL Lo-bind tubes. Gel spots were washed with water and de-stained twice with 50% acetonitrile/25 mM Ambic (ammonium bicarbonate) and then dehydrated with 100% acetonitrile. Posteriorly, 15 μL of trypsin (Sigma-Aldrich, USA) (0.02 μg/μL in Ambic 12.5 mM/2% acetonitrile) was added to the gel spots and incubated for 60 min on ice. Then, the gels were covered with 25–50 μL of 12.5 mM Ambic depending on the spot volume. The samples were incubated for 18 h, overnight at 37°C. Tubes were chilled to room temperature, the gel pieces spin down and the supernatants collected to a new tube. Then 25 μL formic acid 5% (v/v) were added to the remaining gel pieces, vortexed and incubated for 15 min at 37°C. The supernatants were collected once again to the tubes and 25 μL of 50% ACN/ 0.1% TFA was added. Once more the supernatant was collected to the first tube, solution dried-down in SpeedVac (Thermo Fisher Scientific Waltham, MA, USA) and the dried peptides stored at −20°C until MS and MS/MS analyses.

#### Tandem mass spectrometry

Tryptic peptides were re-suspended in 50% acetonitrile/0.1% formic acid solution and mixed (1:1) with a matrix consisting of a saturated solution of a-cyano-4-hydroxycinnamic acid prepared in 50% acetonitrile/ 0.1% trifluoracetic acid. Three aliquots of samples (0.5 μL) were spotted onto the MALDI sample target plate. Peptide mass spectra were obtained on a MALDI-TOF/TOF mass spectrometer (4800 Proteomics Analyzer, Applied Biosystems, Europe) in the positive ion reflector mode. Spectra were obtained in the mass range between 900 and 4,500 Da with ca. 1,500 laser shots. For each sample spot, a data dependent acquisition method was created to select the six most intense peaks, excluding those from the matrix, or acrylamide peaks, for subsequent MS/MS data acquisition.

#### Database search

Spectra were processed and analyzed by the Global Protein Server Workstation (Applied Biosystems), which uses internal MASCOT software (v2.1.0 Matrix Science, London, UK) on searching the peptide mass fingerprints and MS/MS data. Either NCBI *S. aurata* database or Swiss-Prot non-redundant protein sequence database (October 2014) under the taxonomy Chordata were used for searches. Database search parameters were as follows: carbamidomethylation and propionamide of cysteine as a variable modification as well as oxidation of methionine, and the allowance for up to two missed tryptic cleavages. The peptide mass tolerance was 40 ppm and fragment ion mass tolerance was 0.3 Da. Positive identifications were accepted up to 95% of confidence level (MASCOT score >60, calculated as −10 × log *P*, where P is the probability that the observed match is a random event; this is the lowest score indicated by the program as significant, *P* < 0.05, and indicated by the probability of incorrect protein identification). Highest MASCOT score and peptide representativity within prospected taxa were taken as main matching criteria (e.g., in a few spots, the MASCOT search retrieved several significant matches but these represented the same protein, in different species; in these cases, the species with the highest score was chosen).

#### Cluster analysis and pathway network

A two-way hierarchical clustering analysis was carried out using Cluster 3.0 (Stanford University, CA, USA) plus Java TreeView (Saldanha, 2004). Normalized (spot volume/total spot volume of all proteins in a gel image) average spot volumes were used for this analysis, which followed the criteria (i) adjustments to data: log transformation, center genes (mean), normalize genes (row-wise standardized expression values) (ii) hierarchical analysis: cluster genes and arrays, (iii) similarity metrics: correlation (uncentered) for genes and Euclidean distance for arrays, (iv) clustering method: complete linkage. Moreover, a Venn diagram was constructed (using open source Venny 2.0, http://bioinfogp.cnb.csic.es/tools/venny/index.html, BioinfoGP, National Biotechnology Centre, Spain) to illustrate shared and exclusively regulated proteins between fish collected at 24 and 30°C after 14 and 21 days of exposure to warming. A protein network analysis was also performed using open source ClueGo+CluePedia 2.1.7 plugin (from Cytoscape v3.2 platform), to depict interactions between the differentially expressed proteins after 14 and 21 days of exposure to high temperatures. The analysis was performed using the following criteria: species *Homo sapiens*; gene ontology settings KEGG 05.05.2015, WikiPathways 05.05.2015, GO ImmuneSystemProcess 30.04.2015_07h53, REACTOME 05.05.2015, GO BiologicalProcess 30.04.2015_07h53; GO Tree interval from 3 to 8; GO term pathway selection includes at least 1 gene *per* cluster; kappa score 0.4.; statistical test: right-sided hypergeometric test with Bonferroni step-down correction; significant pathways (*p* ≤ 0.05). All proteins were inserted into the analysis (using human homolog accession numbers) and divided by groups (i) regulated from 18 to 24°C at 14 days (P60174, P11216), (ii) regulated from 18 to 30°C at 14 days (P06733, P06732, P60174, P0DMV8, P11142, P00568, P62736, P06732, P00915), (iii) regulated from 24 to 30°C at 14 days (P50395, P29120, P06732, P11142, P0DMV8, P00568, Q14240, P06732, P00915), (iv) regulated from 18 to 24°C at 21 days (Q8IVG5, P00568, P06744), (v) regulated from 18 to 30°C at 21 days (P0DMV8, P00568, P11142, P04406, P0DMV9), (vi) regulated from 24 to 30°C at 21 days (P0DMV8, P00568, P11142, P60174, P04406, Q8IVG5, P0DMV9, P06744, P00367). Based on these parameters the output network included the proteins creatine kinase M-type, glucose-6-phosphate isomerase, triose phosphate isomerase, glutamate dehydrogenase, carbonic anhydrase, heat shock cognate 71 kDa, eukaryotic initiation factor 4A2, glyceraldehyde-3-phosphate dehydrogenase, alpha-enolase, and glycogen phosphorylase.

#### Categorization of identified proteins into functional classes

Protein function was categorized using open source gene ontology tools, namely UniProt, GeneCards, neXtprot beta, InterPro, and Qiagen—Gene Globe Pathways.

#### Validation of proteomic results

The heat shock protein 70 kDa was chosen as target for validation via Enzyme Linked Immunosorbent Assay, following the method described in Madeira et al. (2014), using anti-hsp70 (Acris, Germany) as primary antibody (1 μg.mL^−1^) and anti-mouse IgG, fab specific, alkaline phosphatase conjugate (Sigma-Aldrich) as secondary antibody (1 μg.mL^−1^). Data were normalized against total protein calculated via the Bradford method, as described in Madeira et al. (2014). Non-parametric Kruskal–Wallis (as the data did not meet both assumptions of normality and homoscedasticity) followed by multiple comparisons of mean ranks were carried out to detect significant differences between Hsp70 levels at different temperatures at 14 and 21 days of exposure. Statistics were carried out in STATISTICA (StatSoft, v10).

## Results

### Assessment of thermal environments

Water temperatures in the Portuguese coastal area range approximately from 10 to 16°C during the winter and 15 to 20°C during the summer. Temperatures are usually lowest in February (average of 13.9°C, minimum of 12.2°C and maximum of 15.2°C) and highest in September (average of 19.3°C, minimum of 17.2°C and maximum of 21.8°C). Coastal water temperatures predicted for 2100 (based on a 2–3°C increase—Miranda et al., 2002) are in the range of 13–19°C and 18–23°C during the winter and summer, respectively. Nevertheless, such temperatures can also occur in present days for a limited period of time (days to weeks; see Madeira et al., 2012). Estuarine temperatures range approximately from 10 to 12°C during the winter to 20-24°C during summer. During heat waves (at least 5 days in a row with a maximum air temperature of ≥ 35°C—Santos and Miranda, 2006), estuaries can reach maximum temperatures between 25 and 28°C, and persisting for over 2 weeks (e.g., see Costa, 1990; Azevedo et al., 2006; Cabral et al., 2007; Madeira et al., 2012). Following the scenario of a 2–3°C increase in Portuguese waters by 2100 (Miranda et al., 2002), estuaries' mean temperature during summer would be in the range of 22–27°C, reaching 30°C during heat waves.

### Fitness assessment

#### Mortality rates and Fulton's K condition index

**Figure 1:**
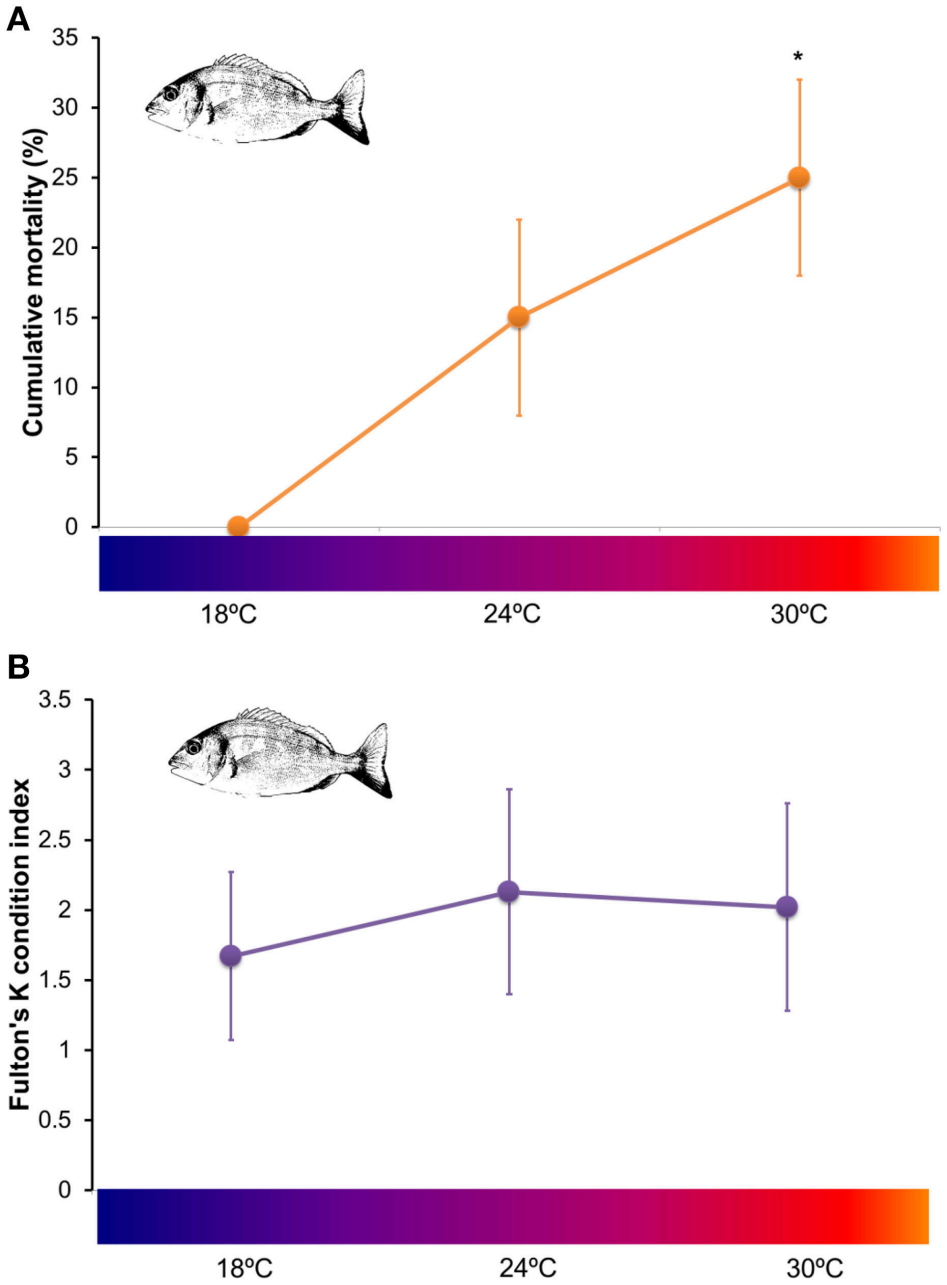
Fitness assessment of *Sparus aurata* juveniles exposed to 18, 24, and 30°C (2 tanks per temperature) **(A)** Cumulative mortalities calculated per tank (mean ± *SD*) after 28 days of exposure (analyzed through Student's *t*-tests, applying Bonferroni correction, and significance level of 0.05). Asterisks mark significant differences from the control group (18°C); **(B)** physiological condition (measured through Fulton's K condition index; mean ± *SD* of 4 individuals in each temperature, 2 from each tank) of juvenile *Sparus aurata* after 21 days of exposure to 18, 24, and 30°C (data was compared via a Kruskal–Wallis test, significance level of 0.05). No significant differences were detected for Fulton's K index among treatments.

At the end of the experimental period no mortality was registered for *S. aurata* juveniles exposed to 18°C. However, a cumulative mortality of 15 ± 7% was observed for fish exposed to 24°C and 25 ± 7% for fish exposed to 30°C (Figure 1A). No significant differences were detected between cumulative mortalities at 18 and 24°C (*t* = −3; *df* = 2; *p* = 0.095). Nevertheless, juveniles exposed to 24°C started dying after 26 days of exposure whereas juveniles exposed to 30°C started dying after 25 days of exposure. Statistical analysis detected significant differences between cumulative mortality at 18°C and 30°C (*t* = −5; *df* = 2; *p* = 0.037). Fulton's K condition index calculated at 21 days of exposure did not vary between fish subjected to 18, 24 and 30°C (Kruskal–Wallis, *H* = 1.28, *p* = 0.526; Figure 1B).

### Proteome modulation

**Figure 2:**
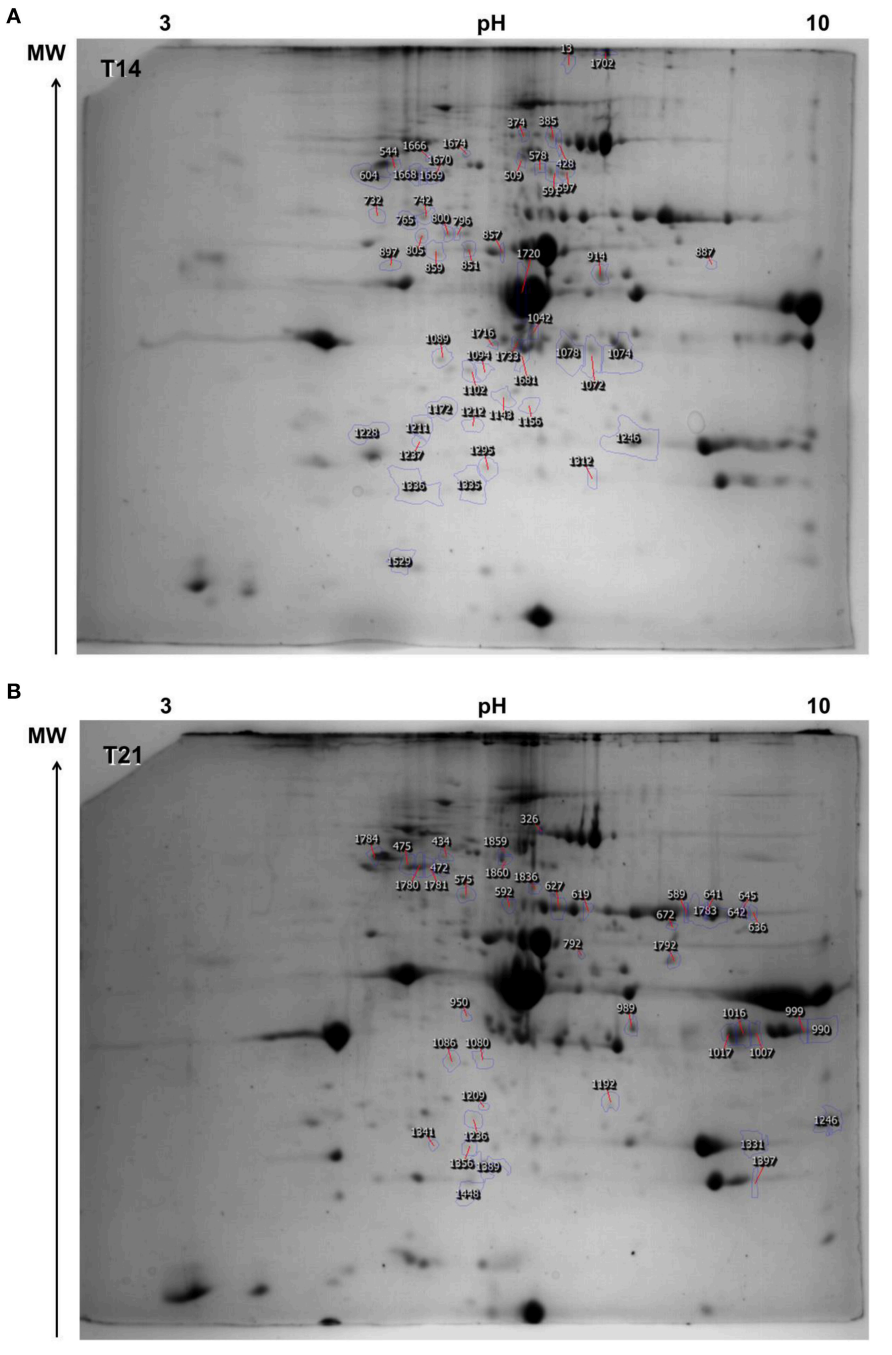
Representative image of the master gel depicting the protein spots detected in *Sparus aurata* juveniles. Annotated spots were those that were differentially expressed between temperature groups (18, 24, and 30°C; *n* = 4 individuals in each group, 2 individuals per tank; 2 technical replicates (gels) per individual; ANOVA *p* < 0.05) **(A)** time-point 14 days of exposure (T14) and **(B)** time-point 21 days of exposure (T21).

After 14 days of exposure to 18, 24, and 30°C, the ANOVA showed that 52 spots (out of 1,627 originally detected by the software) were differentially abundant between temperature groups (*p* < 0.05; Figure 2A and Tables S1, S2), of which 21 were successfully identified (40.4%; Table 1 and Table S3). Of these, seven were unique proteins (no isoforms). The overall changes in proteome were divided into four clusters, according to expression profile (i) cluster 1—proteins were up-regulated with temperature, especially at 30°C, (ii) cluster 2—proteins were slightly down-regulated at 24°C and strongly up-regulated at 30°C, (iii) cluster 3—proteins were down-regulated at 24°C and similar to control levels at 30°C, (iv) cluster 4—proteins were down-regulated with temperature, especially at 30°C.

**Table 1:** Spots differentially expressed between temperature treatments at 14 days of exposure in the muscle of juvenile *Sparus aurata*.

**Spot no**.	**Change**	**Protein Name**	**Species**	**Accession number**	**Protein MW**	**Protein PI**	**Peptide Count**	**Protein Score**	**Protein Score C.I. %**
**859**	↑24vs30°C	Rab GDP dissociation inhibitor beta	*Canis familiaris*	GDIB_CANFA	50289.74	6.11	2	111	100
**1529**	↑24vs30°C	Neuroendocrine convertase 1	*Homo sapiens*	NEC1_HUMAN	84099.28	5.66	5	64	96.67
**851**	↑18vs30°C	Alpha-enolase	*Xenopus laevis*	ENOA_XENLA	47474.28	5.92	12	159	100
**1733**	↑18vs30°C ↑24vs30°C	Creatine kinase M-type	*Bos taurus*	KCRM_BOVIN	42961.82	6.63	7	132	100
**1246**	↑18vs24°C ↑18vs30°C	Triosephosphate isomerase B	*Danio rerio*	TPISB_DANRE	26810.80	6.45	8	138	100
**1042**	↑18vs30°C ↑24vs30°C	Creatine kinase M-type	*Homo sapiens*	KCRM_HUMAN	43073.89	6.77	5	78.9	99.89
**1720**	↓18vs30°C ↓24vs30°C	Creatine kinase M-type	*Homo sapiens*	KCRM_HUMAN	43073.89	6.77	10	213	100
**1716**	↑18vs30°C ↑24vs30°C	Creatine kinase M-type	*Sus scrofa*	KCRM_PIG	43031.85	6.61	14	78.1	99.87
**1670**	↑18vs30°C ↑24vs30°C	Heat shock 70 kDa protein 1	*Oryzias latipes*	HSP71_ORYLA	70307.01	5.47	16	370	100
**1668**	↑18vs30°C ↑24vs30°C	Heat shock cognate 71 kDa protein	*Cricetulus griseus*	HSP7C_CRIGR	70761.12	5.24	3	87.6	99.98
**1312**	↑18vs30°C ↑24vs30°C	Adenylate kinase isoenzyme 1	*Cyprinus carpio*	KAD1_CYPCA	21475.34	6.64	2	90.4	99.99
**1681**	↑18vs30°C ↑24vs30°C	Creatine kinase M-type	*Canis familiaris*	KCRM_CANFA	43125.89	6.77	5	126	100
**1295**	↑18vs30°C	Adenylate kinase isoenzyme 1	*Cyprinus carpio*	KAD1_CYPCA	21475.34	6.64	4	88.5	99.98
**857**	↑18vs30°C	Alpha-enolase	*Pongo abelii*	ENOA_PONAB	47167.36	7.57	10	108	100
**1674**	↑18vs30°C	Actin, alpha cardiac muscle 2	*Xenopus tropicalis*	ACT2_XENTR	42005.89	5.23	9	115	100
**428**	↓18vs24°C	Glycogen phosphorylase, brain form	*Ovis aries*	PYGB_SHEEP	96253.39	6.57	10	93.9	99.99
**897**	↑24vs30°C	Eukaryotic initiation factor 4A-II	*Macaca fascicularis*	IF4A2_MACFA	46387.79	5.4	12	146	100
**509**	↑24vs30°C	Creatine kinase M-type	*Homo sapiens*	KCRM_HUMAN	43073.89	6.77	8	156	100
**1143**	↑18vs30°C ↑24vs30°C	Creatine kinase, testis isozyme	*Oncorhynchus mykiss*	KCRT_ONCMY	42976.76	6.2	3	57.5	85.14
**1211**	↑18vs30°C ↑24vs30°C	Carbonic anhydrase 1	*Chionodraco hamatus*	CAH1_CHIHA	28324.92	5.58	2	106	100
**1669**	↑18vs30°C ↑24vs30°C	Heat shock 70 kDa protein 1	*Oryzias latipes*	HSP71_ORYLA	70307.01	5.47	11	149	100

**Figure 3:**
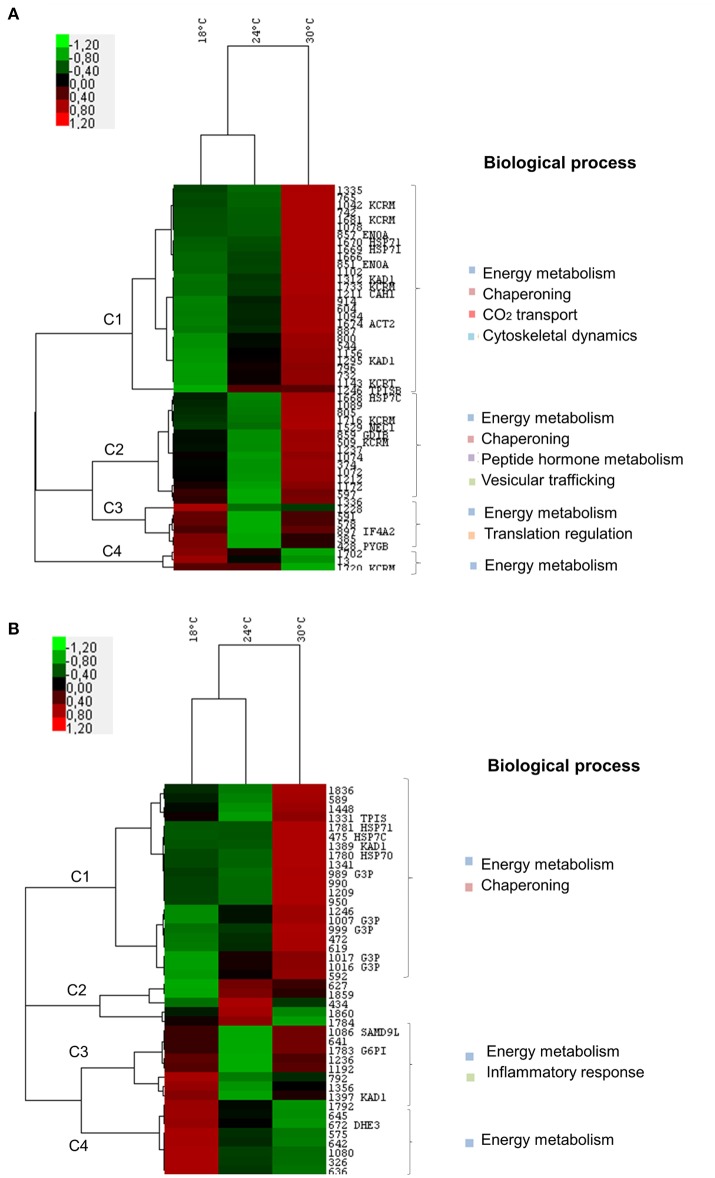
Two-way hierarchical clustering analysis and functional categorization of proteome data from *Sparus aurata* juveniles subjected to 18, 24, and 30°C. Heat map representation of the clustered data matrix in which cells denote the Log2 values of protein normalized volumes. The color scale ranges from green (lower than mean expression level) to red (higher than mean expression level). Columns represent different temperatures while rows represent different proteins. Biological functions were listed for identified proteins in each cluster. **(A)** Time-point: 14 days of exposure. Two proteins were significantly regulated at 24°C i.e., up-regulation of TPISB and down-regulation of PYGB. At 30°C, all identified proteins were significantly up-regulated when compared to 18 and/or 24°C with the exception of PYGB, which returned to control levels and one isoform of KCR, which was significantly down-regulated at 30°C. **(B)** Time-point: 21 days of exposure. Three proteins showed significant differences in expression levels between 18 and 24°C (down-regulation of G6PI, one isoform of KAD1, and of SAMD9L). At 30°C, these proteins were significantly up-regulated in comparison to 24°C. Additionally, all of the other identified proteins were significantly up-regulated at 30°C, when compared to 18 and/or 24°C (see Table S1 for Tukey's *post-hocs*). HSP71, Heat shock 70 kDa protein 1; ENOA, Alpha-enolase; KAD1, Adenylate kinase isoenzyme 1; KCRM, Creatine kinase M-type; CAH1, Carbonic anhydrase 1; ACT2, Actin, alpha cardiac muscle 2; HSP7C, Heat shock cognate 71 kDa protein; GDIB, Rab GDP dissociation inhibitor beta; NEC1, Neuroendocrine convertase 1; TPISB, Triosephosphate isomerase B; KCRT, Creatine kinase, testis isozyme; IF4A2, Eukaryotic initiation factor 4A-II; PYGB, Glycogen phosphorylase; HSP70 - Heat shock 70 kDa protein; G3P, Glyceraldehyde-3-phosphate dehydrogenase; SAMD9L, Sterile alpha motif domain-containing protein 9-like; G6PI, Glucose-6-phosphate isomerase; TPIS, Triosephosphate isomerase (Fragments); DHE3, Glutamate dehydrogenase, mitochondrial.

*Protein level changes are shown in Figure 3A. Arrows indicate significant up-regulation (↑) or down-regulation (↓) between temperature groups*.

After 21 days of exposure, 42 spots (out of the 1,534 detected by the software) were differentially expressed and of these, 14 were successfully identified (33.3%; Figure 2B, Table 2, and Tables S1, S3, S4). Of these, four were unique proteins (no isoforms). The overall changes in proteome were divided into four clusters according to expression profile (i) cluster 1—proteins were up-regulated with temperature, especially at 30°C, (ii) cluster 2—proteins were up-regulated at 24°C and returned to control levels at 30°C, (iii) cluster 3—proteins were down-regulated at 24°C and similar to control levels at 30°C, (iv) cluster 4—proteins were down-regulated at 24 and 30°C.

**Table 2:** Spots differentially expressed between temperature treatments at 21 days of exposure in the muscle of juvenile *Sparus aurata*.

**Spot no**.	**Change**	**Protein Name**	**Species**	**Accession number**	**Protein MW**	**Protein PI**	**Peptide Count**	**Protein Score**	**Protein Score C.I. %**
**1781**	↑18vs30°C ↑24vs30°C	Heat shock 70kDa protein 1	*Oryzias latipes*	HSP71_ORYLA	70307.00	5.47	9	171	100
**1389**	↑18vs30°C ↑24vs30°C	Adenylate kinase isoenzyme 1	*Cyprinus carpio*	KAD1_CYPCA	21475.34	6.64	2	91.8	99.99
**475**	↑18vs30°C ↑24vs30°C	Heat shock cognate 71 kDa protein	*Ictalurus punctatus*	HSP7C_ICTPU	71296.14	5.19	12	148	100
**1331**	↑24vs30°C	Triosephosphate isomerase (Fragments)	*Mesocricetus auratus*	TPIS_MESAU	20294.37	5.49	1	83.5	99.96
**1017**	↑18vs30°C	Glyceraldehyde-3-phosphate dehydrogenase	*Danio rerio*	G3P_DANRE	35761.30	8.2	3	62.4	95.19
**989**	↑18vs30°C ↑24vs30°C	Glyceraldehyde-3-phosphate dehydrogenase (Fragment)	*Meleagris gallopavo*	G3P_MELGA	24836.69	7.22	3	123	100
**1086**	↓18vs24°C ↑24vs30°C	Sterile alpha motif domain-containing protein 9-like	*Homo sapiens*	SAM9L_HUMAN	184415.39	8.25	8	67.4	98.48
**999**	↑18vs30°C ↑24vs30°C	Glyceraldehyde-3-phosphate dehydrogenase	*Danio rerio*	G3P_DANRE	35761.30	8.2	2	163	100
**1007**	↑18vs30°C	Glyceraldehyde-3-phosphate dehydrogenase	*Danio rerio*	G3P_DANRE	35761.30	8.2	2	146	100
**1016**	↑18vs30°C	Glyceraldehyde-3-phosphate dehydrogenase	*Danio rerio*	G3P_DANRE	35761.30	8.2	2	70.4	99.24
**1780**	↑18vs30°C ↑24vs30°C	Heat shock 70 kDa protein	*Oncorhynchus tshawytscha*	HSP70_ONCTS	70931.89	5.42	6	118	100
**1397**	↓18vs24°C	Adenylate kinase isoenzyme 1	*Cyprinus carpio*	KAD1_CYPCA	21475.34	6.64	2	108	100
**1783**	↓18vs24°C ↑24vs30°C	Glucose-6-phosphate isomerase	*Bos taurus*	G6PI_BOVIN	62815.04	7.33	5	75.8	99.78
**672**	↓18vs30°C	Glutamate dehydrogenase, mitochondrial	*Chaenocephalus aceratus*	DHE3_CHAAC	55359.09	7.34	2	84.4	99.97

*Protein level changes are shown in Figure 3B. Arrows indicate significant up-regulation (↑) or down-regulation (↓) between temperature groups*.

Overall the cluster analysis showed that, at both time-points, juvenile fish had more similar expression profiles at 18 and 24°C, while such expression noticeably changed at 30°C (Figures 3A,B). This pattern was mainly linked to cluster 1 at both time-points, in which the main biological processes identified were energy metabolism and chaperoning (common to both time-points) and cytoskeletal dynamics and CO_2_ transport (only at T14).

At both T14 and T21, the significant changes observed between 18 and 24°C were related to energetic metabolism. While at T14, there was an up-regulation of triose phosphate isomerase and a down-regulation of glycogen phosphorylase (Figure 3A; see Table S1 for Tukey's *post-hocs*), at T21 there was a down-regulation of glucose-6-phosphate isomerase and one isoform of adenylate kinase from 18 to 24°C. Moreover, at T21, a protein related to inflammatory processes was down-regulated from 18 to 24°C (sterile alpha motif domain-containing protein 9-like). At 30°C all of these proteins were significantly up-regulated with the exception of glycogen phosphorylase which returned to control levels (Figure 3; see Table S1 for Tukey's *post-hocs*).

Moreover, the other proteins involved in energy balance metabolism were up-regulated at 30°C at both T14 and T21, with the exception of one isoform of creatine kinase at T14 and glutamate dehydrogenase at T21, which were down-regulated. At 30°C, proteins involved in chaperoning (both T14 and T21), CO_2_ transport, cytoskeletal dynamics, peptide hormone metabolism and vesicular trafficking (T14) were also up-regulated (in comparison to 18 and/or 24°C; Figures 3A,B). Proteins involved in translational regulation were also detected at T14, mostly increasing from 24 to 30°C.

**Figure 4:**
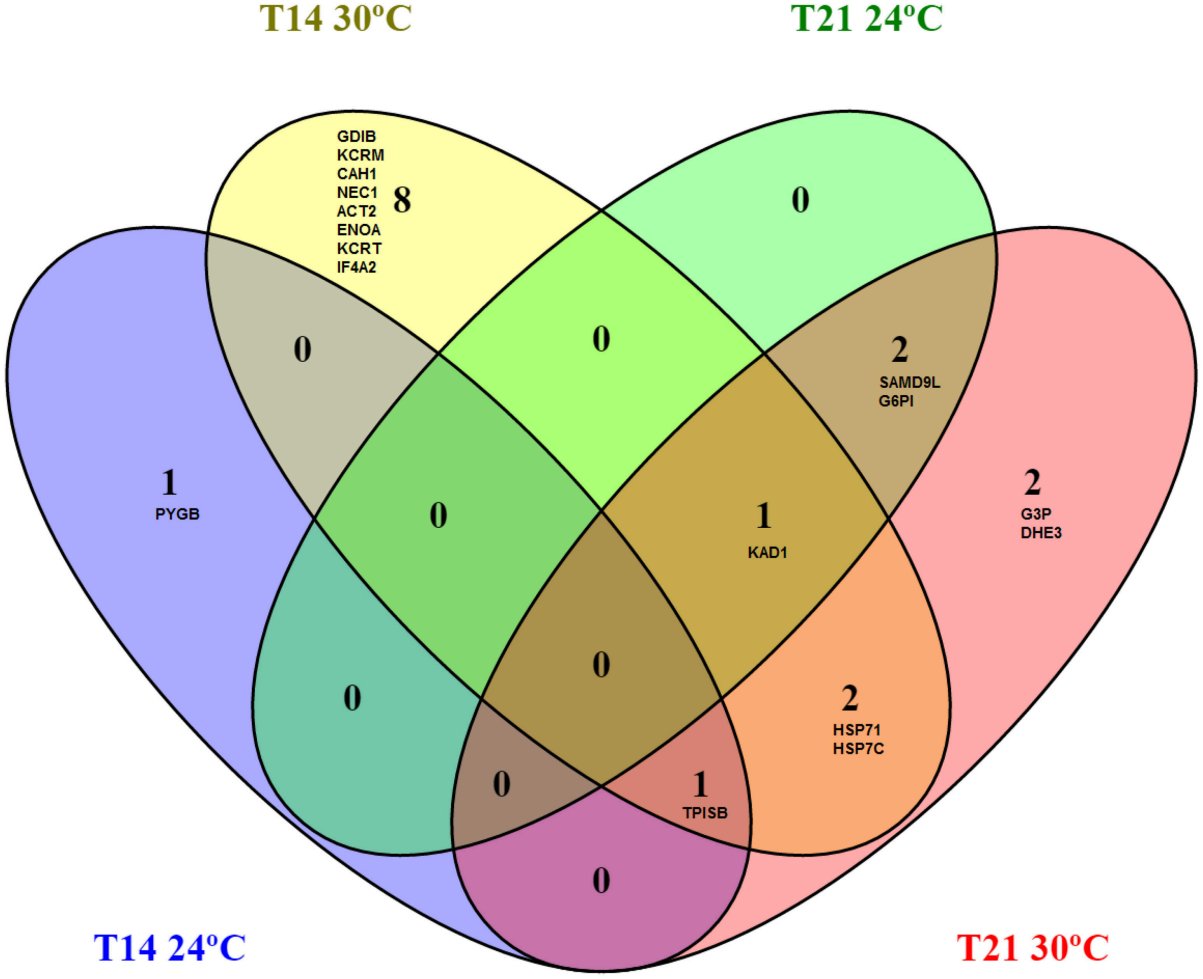
Venn diagram of muscle proteome of *Sparus aurata* collected at 24 and 30°C (after 14 and 21 days of exposure) showing shared and exclusively regulated proteins among temperatures and exposure times. Proteins common to both temperatures were TPISB, KAD1 (up-regulated at 24°C and 30°C), SAMD9L and G6PI (down-regulated at 24°C and up-regulated at 30°C). The four proteins shared between exposure times were HSP71, HSP7C, KAD1, and TPISB (which were all up-regulated at 30°C at both exposure times). All redundancies were eliminated in this analysis. T14, 14 days of exposure; T21, 21 days of exposure; HSP71, Heat shock 70 kDa protein 1; ENOA, Alpha-enolase; KAD1, Adenylate kinase isoenzyme 1; KCRM, Creatine kinase M-type; CAH1, Carbonic anhydrase 1; ACT2, Actin, alpha cardiac muscle 2; HSP7C, Heat shock cognate 71 kDa protein; GDIB, Rab GDP dissociation inhibitor beta; NEC1, Neuroendocrine convertase 1; TPISB, Triosephosphate isomerase B; KCRT, Creatine kinase, testis isozyme; IF4A2, Eukaryotic initiation factor 4A-II; PYGB, Glycogen phosphorylase; HSP70 - Heat shock 70 kDa protein; G3P, Glyceraldehyde-3-phosphate dehydrogenase; SAMD9L, Sterile alpha motif domain-containing protein 9-like; G6PI, Glucose-6-phosphate isomerase; TPIS, Triosephosphate isomerase (Fragments); DHE3, Glutamate dehydrogenase, mitochondrial.

In summary, the majority of proteins regulated after 14 and 21 days of exposure to warming were related to energetic metabolism (61% at T14 and 72% at T21) and chaperoning (14% at T14 and 21% at T21), while other categories were less represented (Figure S2). The Venn diagram showed that proteins common to both temperatures were triosephosphate isomerase, adenylate kinase 1 (up-regulated at 24 and 30°C), sterile alpha motif domain containing protein 9-like and glucose 6 phosphate isomerase (down-regulated at 24°C and up-regulated at 30°C) whereas proteins common to both time-points were heat shock 70 kDa protein 1, heat shock cognate 71 kDa protein, adenylate kinase isoenzyme 1 and triosephosphate isomerase (which were all up-regulated at 30°C at both exposure times; Figure 4).

**Figure 5:**
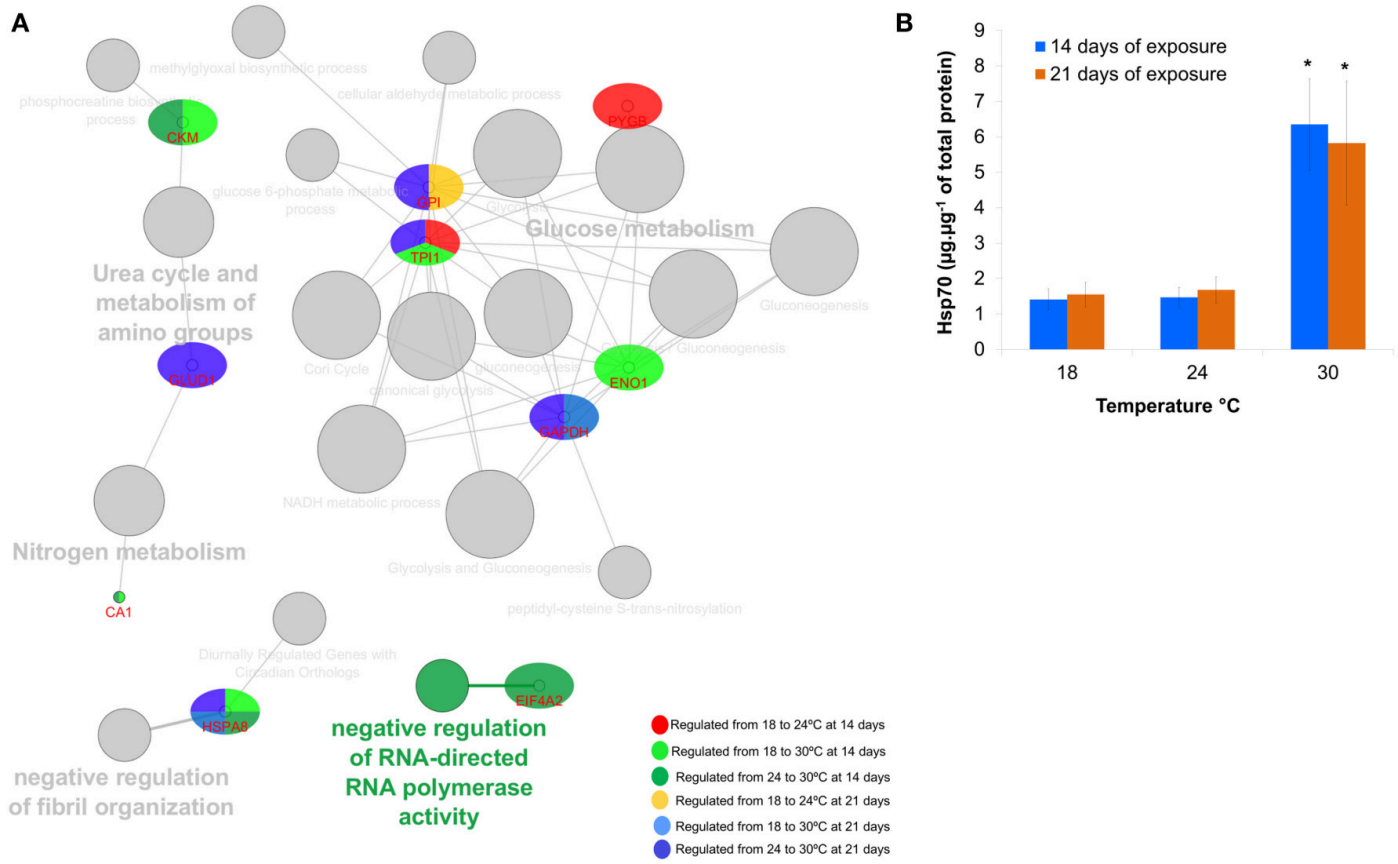
**(A)** Protein network analysis carried out using ClueGo+CluePedia 2.1.7 plugin to depict interactions between the differentially expressed proteins. Node size relates to significance and number of genes associated to that biological process; **(B)** Hsp70 levels (mean ± *sd*) measured by enzyme-linked immune-sorbent assay in the muscle of *Sparus aurata* juveniles exposed to thermal stress for 14 and 21 days.

From the functional protein network, a predominance of glucose metabolism was observed for all groups (Figure 5A), being especially relevant in fish subjected to 24°C at both 14 and 21 days. When fish were exposed to 30°C at 14 days, additional mechanisms besides glucose metabolism seemed relevant including negative regulation of fibril organization, nitrogen metabolism, amino groups metabolism, and phosphocreatine processes. When fish were exposed to 30°C at 21 days (comparing to both 18 and 24°C), glucose metabolism, negative regulation of fibril organization and metabolism of amino groups appear as relevant processes.

#### Validation of proteomic data

Hsp70 levels were significantly different between 18 vs. 30°C and 24 vs. 30°C at both sampling points (14 days of exposure: Kruskal–Wallis test *H* = 9.42, *p* = 0.0090; 21 days of exposure *H* = 9.50, *p* = 0.0087; Figure 5B).

## Discussion

In this study, *S. aurata* juveniles modulated muscle proteins mostly when exposed to 30°C, while fish exposed to 24°C regulated fewer proteins, typically related to energy metabolism. These results coupled to a lack of a cellular stress response at 24°C suggest that this temperature is not very stressful to juvenile fish; alternatively, the cellular stress response could have been induced early on in the exposure period, but if this occurred, the fish were able to repair damage and acclimate as no changes related to the stress response/tissue damage were detected. Either way, *S. aurata* were able to cope with 24°C. This may be explained by the fact that juveniles inhabit low depth waters (i.e., estuaries and coastal lagoons) that can reach such temperatures quite often in the summer (Froese and Pauly, 2006; Madeira et al., 2012). However, 30°C was sufficiently warm to elicit the cellular stress response, indicated by the up-regulation of chaperones (heat shock proteins) and cytoskeletal re-arrangements, corroborating other acute and chronic experiments performed in aquatic animals (including *S. aurata*; Feidantsis et al., 2009; Madeira et al., 2014; Jayasundara et al., 2015; Tomalty et al., 2015).

Chaperones are cytoprotective proteins that stabilize denatured proteins and have a selective value (Hofmann, 2005) since they confer stress tolerance and delay thermal injury (Feder, 1996; Horowitz, 2001; Basu et al., 2002; Sorte and Hofmann, 2005; Bahrndorff et al., 2009). Moreover, several authors have reported a link between molecular chaperones and cytoskeletal components (Liang and MacRae, 1997; Mounier and Arrigo, 2002; Tomanek and Zuzow, 2010), suggesting that cytoskeletal proteins are very sensitive to temperature and chaperones may therefore play a decisive role on their modulation, function, and protection. In fact, cytoskeleton re-modeling may be essential to stabilize cytoskeletal components and maintain homeostatic balance upon challenging conditions (Buckley et al., 2006; Garlick and Robertson, 2007). This may be crucial not only to preserve cellular activities related to motility, signaling, and organelle movement but also to adjust ATP turn-over rates (see Garland et al., 2015) and maintain muscle contraction.

Besides up-regulating the cellular stress response, fish exposed to 30°C had significantly higher mortality rates. Increased mortality induced by temperature has also been observed in larval stages of *S. aurata* (Madeira et al., 2016) and throughout the life cycle of other fish species (e.g., Houde, 1989; Hinch and Martins, 2011). However, thermotolerance, survival and the potential for acclimation of each individual may depend on several factors such as thermal history, sex, nutritional condition, diseases, age and reproductive status (e.g., Becker and Genoway, 1979; Lutterschmidt and Hutchison, 1997; Underwood et al., 2012; Bruneaux et al., 2014). In accordance with higher mortality levels, fish exposed to 30°C presented signs of an inflammatory process or changed immune response, indicated by the up-regulation of sterile alpha motif domain-containing protein 9-like (from 24 to 30°C). Thermal stress is known to cause damage to cellular components and tissues in aquatic organisms, including *S. aurata* (e.g., Madeira et al., 2014, 2015; Raina et al., 2015). As such, proteins or transcripts involved in the inflammatory process and immune response are commonly regulated upon exposure to thermal stress (e.g., Thorne et al., 2010; Windisch et al., 2014; Basu et al., 2015; Garland et al., 2015; Tomalty et al., 2015).

Interestingly, no changes were detected in Fulton's K condition index. Stressful environments likely lead to higher energy demands, thus fish subjected to heat stress could have lower condition due to weight loss. However, juvenile fish did not seem to experience significant weight loss, despite the detected changes in energetic metabolism. Alternatively, we can also consider that the exposure time was not enough to observe changes in Fulton's K condition index. Nonetheless, proteins related to energetic pathways were both regulated at 24 and 30°C. Overall, most of these proteins were up-regulated upon warming. This indeed suggests a higher energy demand at higher temperatures, which is in accordance with other studies that show that metabolic rates increase in fish chronically exposed to high temperatures (e.g., Rosa et al., 2014; Vinagre et al., 2014). This is especially relevant in muscle, which has high energetic demands and has to provide energy during long-periods or increase the rate of energy production in response to explosive contractions (Westerblad et al., 2010), in order to maintain locomotion. In fact, adjustments in energy metabolism may be essential for fish to be able to move to thermal refugia or keep their activities of foraging.

Overall, changes in energetic metabolism suggest an enhanced glycolytic potential and increased carbohydrate metabolism mainly at 30°C but also, to some extent, at 24°C. This finding is supported by the up-regulation of alpha-enolase, triose phosphate isomerase and glyceraldehyde-3-phosphate dehydrogenase. Moreover, the up-regulation of creatine kinase isoforms and adenylate kinase indicates the regulation of ATP and AMP levels by producing ATP from phosphocreatine and ADP, and converting 2 ADP ⇆ ATP + AMP, respectively. These reactions contribute to the maintenance of ATP levels and regulate glycolytic enzymes (Berg et al., 2002; Fischer, 2013), being especially relevant in tissues with high energy requirements such as skeletal muscle. Its functional capacity depends on large energy budgets (Kenyon and Reed, 1983; Wallimann et al., 1992), which could hardly be sustained by the low pools of cellular ATP. Alternatively, phosphagens are accumulated in higher concentrations and can thus be used to produce energy and replenish ATP levels maintaining energy homeostasis (Wallimann et al., 1992). Although, most isoforms of creatine and adenylate kinases were up-regulated with temperature, there was also two down-regulated isoforms. The functional explanation for this opposed expression pattern is not clear but it may depend on the isoenzyme-specific subcellular localization and bounding properties, leading to slightly different physiological outcomes related to energetic metabolism and cellular structural properties (e.g., see Hornemann et al., 2000). However, as it was not possible to determine which specific isoenzymes were regulated, we cannot take further conclusions.

Changes in glycolytic enzymes have also been detected in other studies of acute (Garland et al., 2015) and chronic exposure to temperature (Jayasundara et al., 2015), usually increasing at higher temperatures. However, Garland et al. (2015) also detected that such enzymes could undergo antagonist changes, with enzymes at the preparatory phase decreasing and enzymes involved in the pay-off phase increasing. Moreover, there seemed to be a decrease in glycogenolysis at 24°C after 14 days of exposure indicated by the down-regulation of glycogen phosphorylase. This suggests that fish can maintain some glycogen reserves if the temperature increase is mild. After 21 days of exposure, glucose-6-phosphate isomerase was also down-regulated at 24°C suggesting a decrease in the inter-conversion of glucose-6-phosphate to fructose-6-phosphate (glycolysis/gluconeogenesis). This indicates that after 21 days of exposure to 24°C there is no longer the need for an enhanced glycolytic potential which points out that fish may be acclimating to this temperature. However, after 21 days of exposure to 30°C there seemed to be a decline in the tricarboxylic acid cycle (or related anaplerotic reactions), possibly suggesting a decrease in aerobic metabolism, since this cycle is dependent on the oxidation of acetyl-CoA and produces NADH to fuel the oxidative phosphorylation pathway. The potential decline in this pathway is supported by the down-regulation of glutamate dehydrogenase, which produces intermediate substrates for the tricarboxylic acid cycle (alpha ketoglutarate). Some studies suggest that exposure to high temperature induces a metabolic shift toward carbohydrate metabolism and the formation of glycogen stores (Brodte et al., 2006; Windisch et al., 2011, 2014), because these metabolic mechanisms are able to function under hypoxaemia, which is generally induced by hyperthermia (oxygen limited thermal tolerance, Pörtner and Farrell, 2008). Entering a state of hypoxaemia means a shift from aerobic to anaerobic metabolism at some point and this threshold for anaerobiosis is dependent on factors such as species and acclimation history (e.g., see Burleson and Silva, 2011). However, in this case, it is not entirely clear if pyruvate obtained from increased glycolysis is being used to maintain aerobic metabolism or is being converted to lactate in anaerobic conditions. Still, the down-regulation of glutamate dehydrogenase suggests that long-exposure to extreme temperatures decreases aerobic metabolism, potentially eliciting a transition to anaerobic metabolism.

Physiological adjustments to stress were influenced not only by temperature but also by the duration of exposure. While some mechanisms activated were common to both time-points (e.g., chaperoning), there seemed to be a transition in the predominant processes of response from T14 to T21. At T14, transcriptional/translational regulation, amino acid homeostasis and acid-base balance seemed more relevant while at T21, the shifting of the energetic metabolism was predominant and related to gluconeogenesis and cellular carbohydrate biosynthetic processes, which may be crucial to sustain metabolic performance under chronic stress.

Indeed, at T14, exposure to extreme temperature seems to have elicited the activation of endocrine processes and these may be responsible for the activation of molecular adaptive cascades, in which vesicular trafficking and translational regulation could play an important role. This finding is supported by the up-regulation of neuroendocrine convertase 1, eukaryotic initiation factor 4A-II and Rab GDP dissociation inhibitor beta in the muscle of juvenile fish exposed to 30°C. Several studies have shown that a number of hormones dictate responses to stress in fish and other organisms (e.g., Moalic et al., 1989; Barton and Iwama, 1991; Iwama et al., 1999; Barton, 2002; Pepels et al., 2004) and that modulation of translation and transcription is crucial to deal with acute and chronic thermal stress in fish (e.g., Long et al., 2012; Windisch et al., 2014; Tomalty et al., 2015). Moreover, the regulation of acid-base status may be an important mechanism for homeostatic balance in fish as suggested by the up-regulation of carbonic anhydrase 1 at T14 (30°C). However, the influence of hyperthermia in acid-base balance in fish is not well-known, as opposed to the effect of hypothermia (Pörtner et al., 1998) and other environmental factors such as salinity and pollutants (e.g., de Polo et al., 2014).

## Conclusion

**Figure 6:**
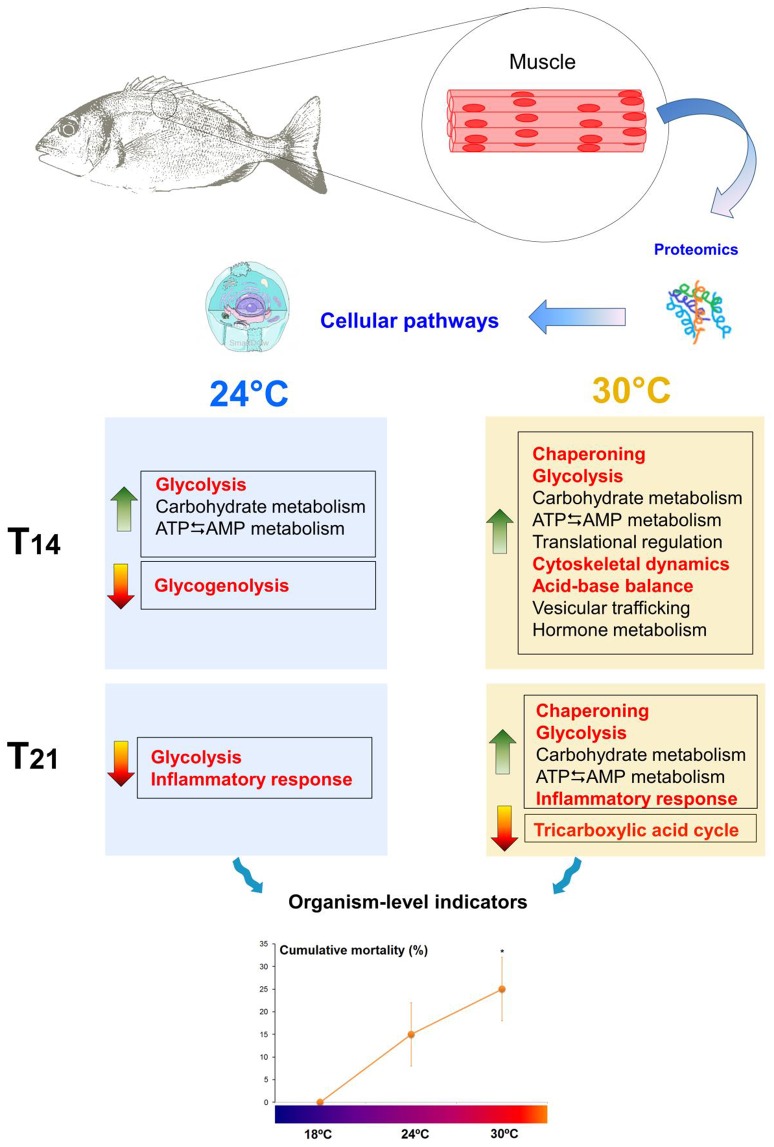
Summary of the study. *Sparus aurata* juveniles exposed to warm temperatures (control 18°C vs. 24°C and 30°C) modulated muscle proteins related to energetic processes, chaperoning, cytoskeletal dynamics, acid-base balance, peptide hormone metabolism, vesicular trafficking and inflammatory processes. Fish seemed more able to acclimate to 24°C, as opposed to 30°C, as shown by significantly higher levels of cumulative mortality at this temperature. The mechanisms hypothesized to have the greatest influence on fitness outcomes are highlighted in red. Asterisks indicate significant differences from control (*p* < 0.05). Green arrows indicate up-regulation (↑) and red arrows indicate down-regulation (↓). T14, 14 days of exposure; T21, 21 days of exposure. *S. aurata* drawings by D. Madeira; cell image from SmartDraw software, LifeART Collection Images Copyright 1989-2001 by Lippincott Williams & Wilkins, Baltimore, MD.

Molecular information, coupled with performance measurements, reflects the health status of fish and their ability to successfully acclimate to new environmental conditions. In this study, sea bream juveniles mildly responded to 24°C but showed a stronger response to 30°C. While exposure to 24°C only elicited energetic adjustments (higher glycolytic potential and regulation of ATP levels, maintenance of glycogen reserves), 30°C seems to induce much more molecular changes. At this temperature, the maturation of peptide hormones probably led to the activation of molecular cascades which potentially triggered energetic adjustments (enhanced glycolytic potential and regulation of ATP levels) necessary to induce the cellular stress response (increased chaperoning, cytoskeletal re-arrangements, and regulation of acid-base balance). Such changes are known to contribute to cellular homeostasis and muscle functioning. Nevertheless, the data indicate that aerobic metabolism may be compromised (down-regulation of the tricarboxylic acid cycle) and an inflammatory process could be ongoing in animals subjected to 30°C, which probably contributed to higher levels of mortality (Figure 6—summary figure). Overall, the molecular information coupled with mortality rates suggests that juvenile fish are able to acclimate to 24°C, but may be vulnerable to temperatures above 24°C and may not be able to fully acclimate to 30°C. *S. aurata* is an eurythermal subtropical species (with an upper thermal limit of 35.5°C—Madeira et al., 2014) that inhabits shallow habitats, in which a temperature of 24°C is fairly common during summer. However, 30°C (predicted future heat wave condition in Portuguese estuaries) seems somewhat deleterious to these sea breams suggesting that their health may be hindered if heat waves increase in frequency and intensity, as predicted by IPCC (2013). Therefore, recruitment and population levels of *S. aurata* may be affected by ocean warming, highlighting the need for improvement of management plans for fish stocks (e.g., limiting catch quotas as needed). Nevertheless, the ecological consequences of a warming ocean are dependent on several factors, including phenotypic plasticity throughout ontogeny, local adaptation (and thus larval dispersal), transgerational plasticity (e.g., Munday, 2014), and trophic-web cascading effects (e.g., Nagelkerken and Connell, 2015), warranting further research. Moreover, regardless of the enormous advances in proteomics and the significant findings of the present study, this approach still presents some limitations. Thus, for future studies a systems biology approach can be used to provide more information.

## Data availability

Accession numbers: GDIB_CANFA; NEC1_HUMAN; ENOA_XENLA; KCRM_BOVIN; TPISB_DANRE; KCRM_HUMAN; KCRM_PIG; HSP71_ORYLA; HSP7C_CRIGR; KAD1_CYPCA; KCRM_CANFA; ENOA_PONAB; ACT2_XENTR; PYGB_SHEEP; IF4A2_MACFA; KCRT_ONCMY; CAH1_CHIHA; HSP7C_ICTPU; TPIS_MESAU; G3P_DANRE; G3P_MELGA; SAM9L_HUMAN; HSP70_ONCTS; G6PI_BOVIN; DHE3_CHAAC. See supplemental materials for additional information concerning experimental setup, general categories of gene ontology, detailed functional categorization, masses and sequences of peptides obtained for each spot, protein expression levels and Tukey's *post-hocs*.

## Author contributions

MD and CV designed the study; DM and MD carried out the experiments; DM and JA carried out sample preparation and 2-D gels, gel image analyses and protein digestion; RV performed mass spectrometry and protein identification; DM, RV, and PC carried out bioinformatics analyses; JC contributed with reagents and analytical advice; DM wrote the manuscript with relevant inputs from all authors.

### Conflict of interest statement

The authors declare that the research was conducted in the absence of any commercial or financial relationships that could be construed as a potential conflict of interest.
