# Supplementary material for: Molecular Plasticity under Ocean Warming: Proteomics and Fitness Data Provides Clues for a Better Understanding of the Thermal Tolerance in Fish
Source: Front Physiol. 2017 Oct 23;8:825. doi: 10.3389/fphys.2017.00825 (PMC5660107; doi:10.3389/fphys.2017.00825)

Supplementary information

Authors: Madeira D, Araújo JE, Vitorino R, Costa PM, Capelo JL, Vinagre C, Diniz MS

Title: Molecular plasticity under ocean warming: proteomics and fitness data provides clues for a better understanding of the thermal tolerance in fish

Table S1. Tukey's Post-hocs **a)** 14 days of exposure, **b)** 21 days of exposure.

a) 14 days of exposure **p values ≤ 0.05 are marked with red**

Spot	Protein

1670 Heat shock 70 kDa protein 1
Tukey HSD Post-hoc Test...
18ºC vs 24ºC: Diff=0.0200, 95%CI=-0.0812 to 0.1212, p=0.87
18ºC vs 30ºC: Diff=0.5000, 95%CI=0.3988 to 0.6012, p=0.000
24ºC vs 30ºC: Diff=0.4800, 95%CI=0.3788 to 0.5812, p=0.000

1669 Heat shock 70 kDa protein 1
Tukey HSD Post-hoc Test...
18ºC vs 24ºC: Diff=0.0300, 95%CI=-0.1292 to 0.1892, p=0.88
18ºC vs 30ºC: Diff=0.5700, 95%CI=0.4108 to 0.7292, p=0.000
24ºC vs 30ºC: Diff=0.5400, 95%CI=0.3808 to 0.6992, p=0.000

859 Rab GDP dissociation inhibitor beta
Tukey HSD Post-hoc Test...
18ºC vs 24ºC: Diff=-0.0700, 95%CI=-0.1542 to 0.0142, p=0.11
18ºC vs 30ºC: Diff=0.0800, 95%CI=-0.0042 to 0.1642, p=0.06
24ºC vs 30ºC: Diff=0.1500, 95%CI=0.0658 to 0.2342, p=0.0006

1668 Heat shock cognate 71 kDa protein
Tukey HSD Post-hoc Test...
18ºC vs 24ºC: Diff=-0.0600, 95%CI=-0.1994 to 0.0794, p=0.53
18ºC vs 30ºC: Diff=0.1500, 95%CI=0.0106 to 0.2894, p=0.03
24ºC vs 30ºC: Diff=0.2100, 95%CI=0.0706 to 0.3494, p=0.003

1312 Adenylate kinase isoenzyme
Tukey HSD Post-hoc Test...
18ºC vs 24ºC: Diff=0.0400, 95%CI=-0.0896 to 0.1696, p=0.72
18ºC vs 30ºC: Diff=0.1800, 95%CI=0.0504 to 0.3096, p=0.006
24ºC vs 30ºC: Diff=0.1400, 95%CI=0.0104 to 0.2696, p=0.03

1681 Creatine kinase M-type
Tukey HSD Post-hoc Test...
18ºC vs 24ºC: Diff=-0.0100, 95%CI=-0.1177 to 0.0977, p=0.97
18ºC vs 30ºC: Diff=0.1200, 95%CI=0.0123 to 0.2277, p=0.03

24ºC vs 30ºC: Diff=0.1300, 95%CI=0.0223 to 0.2377, p=0.02

1733 Creatine kinase M-type
Tukey HSD Post-hoc Test...
18ºC vs 24ºC: Diff=0.0400, 95%CI=-0.0789 to 0.1589, p=0.67
18ºC vs 30ºC: Diff=0.1600, 95%CI=0.0411 to 0.2789, p=0.007
24ºC vs 30ºC: Diff=0.1200, 95%CI=0.0011 to 0.2389, p=0.047

1211 Carbonic anhydrase 1
Tukey HSD Post-hoc Test...
18ºC vs 24ºC: Diff=0.0300, 95%CI=-0.0881 to 0.1481, p=0.79
18ºC vs 30ºC: Diff=0.1500, 95%CI=0.0319 to 0.2681, p=0.01
24ºC vs 30ºC: Diff=0.1200, 95%CI=0.0019 to 0.2381, p=0.046

1716 Creatine kinase M-type
Tukey HSD Post-hoc Test...
18ºC vs 24ºC: Diff=-0.0400, 95%CI=-0.1836 to 0.1036, p=0.76
18ºC vs 30ºC: Diff=0.1400, 95%CI=-0.0036 to 0.2836, p=0.05
24ºC vs 30ºC: Diff=0.1800, 95%CI=0.0364 to 0.3236, p=0.01

509 Creatine kinase M-type
Tukey HSD Post-hoc Test...
18ºC vs 24ºC: Diff=-0.1000, 95%CI=-0.3071 to 0.1071, p=0.45
18ºC vs 30ºC: Diff=0.1500, 95%CI=-0.0571 to 0.3571, p=0.19
24ºC vs 30ºC: Diff=0.2500, 95%CI=0.0429 to 0.4571, p=0.02

1042 Creatine kinase M-type
Tukey HSD Post-hoc Test...
18ºC vs 24ºC: Diff=-0.0400, 95%CI=-0.2470 to 0.1670, p=0.88
18ºC vs 30ºC: Diff=0.2000, 95%CI=-0.0070 to 0.4070, p=0.05
24ºC vs 30ºC: Diff=0.2400, 95%CI=0.0330 to 0.4470, p=0.02

1529 Neuroendocrine convertase 1
Tukey HSD Post-hoc Test...
18ºC vs 24ºC: Diff=-0.0300, 95%CI=-0.1834 to 0.1234, p=0.88
18ºC vs 30ºC: Diff=0.1400, 95%CI=-0.0134 to 0.2934, p=0.07
24ºC vs 30ºC: Diff=0.1700, 95%CI=0.0166 to 0.3234, p=0.03

1295 Adenylate kinase isoenzyme 1
Tukey HSD Post-hoc Test...
18ºC vs 24ºC: Diff=0.1100, 95%CI=-0.0500 to 0.2700, p=0.21
18ºC vs 30ºC: Diff=0.1800, 95%CI=0.0200 to 0.3400, p=0.02
24ºC vs 30ºC: Diff=0.0700, 95%CI=-0.0900 to 0.2300, p=0.52

1720 Creatine kinase M-type
Tukey HSD Post-hoc Test...

18ºC vs 24ºC: Diff=0.0000, 95%CI=-0.0534 to 0.0534, p=NaN
18ºC vs 30ºC: Diff=-0.0600, 95%CI=-0.1134 to -0.0066, p=0.03
24ºC vs 30ºC: Diff=-0.0600, 95%CI=-0.1134 to -0.0066, p=0.03

1246 Triosephosphate isomerase B
Tukey HSD Post-hoc Test...
18ºC vs 24ºC: Diff=0.1000, 95%CI=0.0027 to 0.1973, p=0.043
18ºC vs 30ºC: Diff=0.1000, 95%CI=0.0027 to 0.1973, p=0.043
24ºC vs 30ºC: Diff=0.0000, 95%CI=-0.0973 to 0.0973, p=NaN

1674 Actin, alpha cardiac muscle 2
Tukey HSD Post-hoc Test...
18ºC vs 24ºC: Diff=0.0800, 95%CI=-0.1012 to 0.2612, p=0.52
18ºC vs 30ºC: Diff=0.2100, 95%CI=0.0288 to 0.3912, p=0.02
24ºC vs 30ºC: Diff=0.1300, 95%CI=-0.0512 to 0.3112, p=0.19

857 Alpha-enolase
Tukey HSD Post-hoc Test...
18ºC vs 24ºC: Diff=0.0100, 95%CI=-0.2176 to 0.2376, p=0.99
18ºC vs 30ºC: Diff=0.2200, 95%CI=-0.0076 to 0.4476, p=0.05
24ºC vs 30ºC: Diff=0.2100, 95%CI=-0.0176 to 0.4376, p=0.0739

1143 Creatine kinase, testis isozyme
Tukey HSD Post-hoc Test...
18ºC vs 24ºC: Diff=0.0900, 95%CI=-0.0644 to 0.2444, p=0.33
18ºC vs 30ºC: Diff=0.1600, 95%CI=0.0056 to 0.3144, p=0.04
24ºC vs 30ºC: Diff=0.0700, 95%CI=-0.0844 to 0.2244, p=0.49

897 Eukaryotic initiation factor 4A-II
Tukey HSD Post-hoc Test...
18ºC vs 24ºC: Diff=-0.1300, 95%CI=-0.2690 to 0.0090, p=0.07
18ºC vs 30ºC: Diff=0.0100, 95%CI=-0.1290 to 0.1490, p=0.98
24ºC vs 30ºC: Diff=0.1400, 95%CI=0.0010 to 0.2790, p=0.048

851 Alpha-enolase
Tukey HSD Post-hoc Test...
18ºC vs 24ºC Diff=0.0200, 95%CI=-0.1065 to 0.1465, p=0.91
18ºC vs 30ºC: Diff=0.1300, 95%CI=0.0035 to 0.2565, p=0.04
24ºC vs 30ºC: Diff=0.1100, 95%CI=-0.0165 to 0.2365, p=0.096

428 Glycogen phosphorylase, brain form
Tukey HSD Post-hoc Test...
18ºC vs 24ºC: Diff=-0.2600, 95%CI=-0.4937 to -0.0263, p=0.03
18ºC vs 30ºC: Diff=-0.0600, 95%CI=-0.2937 to 0.1737, p=0.79
24ºC vs 30ºC: Diff=0.2000, 95%CI=-0.0337 to 0.4337, p=0.10

b) 21 days of exposure **p values ≤ 0.05 are marked with red**

Spot	Protein

1781 **Heat shock 70kDa protein**

Tukey HSD Post-hoc Test...

18ºC vs 24ºC: Diff=0.0000, 95%CI=-0.1707 to 0.1707, p=NaN

18ºC vs 30ºC: Diff=0.3800, 95%CI=0.2093 to 0.5507, p=0.000

24ºC vs 30ºC: Diff=0.3800, 95%CI=0.2093 to 0.5507, p=0.000

1780 **Heat shock 70 kDa protein**

Tukey HSD Post-hoc Test...

18ºC vs 24ºC: Diff=0.0100, 95%CI=-0.1728 to 0.1928, p=0.99

18ºC vs 30ºC: Diff=0.3400, 95%CI=0.1572 to 0.5228, p=0.0004

24ºC vs 30ºC: Diff=0.3300, 95%CI=0.1472 to 0.5128, p=0.0005

475 **Heat shock cognate 71 kDa protein**

Tukey HSD Post-hoc Test...

18ºC vs 24ºC: Diff=0.0100, 95%CI=-0.1295 to 0.1495, p=0.98

18ºC vs 30ºC: Diff=0.2000, 95%CI=0.0605 to 0.3395, p=0.005

24ºC vs 30ºC: Diff=0.1900, 95%CI=0.0505 to 0.3295, p=0.007

1086 **Sterile alpha motif domain-containing protein 9-like**

Tukey HSD Post-hoc Test...

18ºC vs 24ºC: Diff=-0.1100, 95%CI=-0.1989 to -0.0211, p=0.01

18ºC vs 30ºC: Diff=0.0200, 95%CI=-0.0689 to 0.1089, p=0.84

24ºC vs 30ºC: Diff=0.1300, 95%CI=0.0411 to 0.2189, p=0.004

1389 **Adenylate kinase isoenzyme 1**

Tukey HSD Post-hoc Test...

18ºC vs 24ºC: Diff=-0.0200, 95%CI=-0.1953 to 0.1553, p=0.96

18ºC vs 30ºC: Diff=0.2200, 95%CI=0.0447 to 0.3953, p=0.01

24ºC vs 30ºC: Diff=0.2400, 95%CI=0.0647 to 0.4153, p=0.007

999 **Glyceraldehyde-3-phosphate dehydrogenase**

Tukey HSD Post-hoc Test...

18ºC vs 24ºC: Diff=0.0700, 95%CI=-0.1993 to 0.3393, p=0.79

18ºC vs 30ºC: Diff=0.3400, 95%CI=0.0707 to 0.6093, p=0.01

24ºC vs 30ºC: Diff=0.2700, 95%CI=0.0007 to 0.5393, p=0.04

1007 **Glyceraldehyde-3-phosphate dehydrogenase**

Tukey HSD Post-hoc Test...

18ºC vs 24ºC: Diff=0.1300, 95%CI=-0.0816 to 0.3416, p=0.29

18ºC vs 30ºC: Diff=0.2900, 95%CI=0.0784 to 0.5016, p=0.006

24ºC vs 30ºC: Diff=0.1600, 95%CI=-0.0516 to 0.3716, p=0.16

1783 Glucose-6-phosphate isomerase

Tukey HSD Post-hoc Test...

18ºC vs 24ºC: Diff=-0.1000, 95%CI=-0.1940 to -0.0060, p=0.03

18ºC vs 30ºC: Diff=0.0200, 95%CI=-0.0740 to 0.1140, p=0.85

24ºC vs 30ºC: Diff=0.1200, 95%CI=0.0260 to 0.2140, p=0.01

989 Glyceraldehyde-3-phosphate dehydrogenase (Fragment)

Tukey HSD Post-hoc Test...

18ºC vs 24ºC: Diff=-0.0300, 95%CI=-0.1957 to 0.1357, p=0.89

18ºC vs 30ºC: Diff=0.1700, 95%CI=0.0043 to 0.3357, p=0.04

24ºC vs 30ºC: Diff=0.2000, 95%CI=0.0343 to 0.3657, p=0.01

1017 Glyceraldehyde-3-phosphate dehydrogenase

Tukey HSD Post-hoc Test...

18ºC vs 24ºC: Diff=0.1500, 95%CI=-0.0324 to 0.3324, p=0.12

18ºC vs 30ºC: Diff=0.2000, 95%CI=0.0176 to 0.3824, p=0.03

24ºC vs 30ºC: Diff=0.0500, 95%CI=-0.1324 to 0.2324, p=0.77

672 Glutamate dehydrogenase, mitochondrial

Tukey HSD Post-hoc Test...

18ºC vs 24ºC: Diff=-0.1500, 95%CI=-0.4113 to 0.1113, p=0.34

18ºC vs 30ºC: Diff=-0.2900, 95%CI=-0.5513 to -0.0287, p=0.03

24ºC vs 30ºC: Diff=-0.1400, 95%CI=-0.4013 to 0.1213, p=0.38

1016 Glyceraldehyde-3-phosphate dehydrogenase

Tukey HSD Post-hoc Test...

18ºC vs 24ºC: Diff=0.1500, 95%CI=-0.0528 to 0.3528, p=0.17

18ºC vs 30ºC: Diff=0.2200, 95%CI=0.0172 to 0.4228, p=0.032

24ºC vs 30ºC: Diff=0.0700, 95%CI=-0.1328 to 0.2728, p=0.66

1331 Triosephosphate isomerase (Fragments)

Tukey HSD Post-hoc Test...

18ºC vs 24ºC: Diff=-0.1400, 95%CI=-0.3996 to 0.1196, p=0.38

18ºC vs 30ºC: Diff=0.1300, 95%CI=-0.1296 to 0.3896, p=0.43

24ºC vs 30ºC: Diff=0.2700, 95%CI=0.0104 to 0.5296, p=0.04

1397 Adenylate kinase isoenzyme 1

Tukey HSD Post-hoc Test...

18ºC vs 24ºC: Diff=-0.2400, 95%CI=-0.4643 to -0.0157, p=0.03

18ºC vs 30ºC: Diff=-0.1100, 95%CI=-0.3343 to 0.1143, p=0.45

24ºC vs 30ºC: Diff=0.1300, 95%CI=-0.0943 to 0.3543, p=0.33

Table S2. Average normalized spot volumes extracted from Same Spots concerning the proteomic analysis carried out in the muscle of *Sparus aurata* exposed to 18ºC, 24ºC and 30ºC for 14 days. Bold lines indicate spots identified through mass spectrometry.

Spot	Identification	Anova (p)	Fold	Average Normalised Volumes		
				18ºC	24ºC	30ºC
**1670**	**Heat shock 70 kDa protein 1**	**7,458e-012**	**3,1**	**1,672e+006**	**1,763e+006**	**5,250e+006**
**1669**	**Heat shock 70 kDa protein 1**	**1,534e-009**	**3,6**	**1,094e+006**	**1,203e+006**	**3,984e+006**
765		8,247e-007	1,6	2,292e+006	2,204e+006	3,496e+006
742		3,977e-004	1,4	1,624e+006	1,594e+006	2,270e+006
**859**	**Rab GDP dissociation inhibitor beta**	**8,210e-004**	**1,4**	**2,685e+006**	**2,299e+006**	**3,299e+006**
800		9,267e-004	1,4	1,025e+006	1,197e+006	1,453e+006
1335		9,464e-004	1,5	5,377e+006	5,081e+006	7,821e+006
1702		0,002	2,4	2,864e+006	2,105e+006	1,187e+006
**1668**	**Heat shock cognate 71 kDa protein**	**0,002**	**1,6**	**3,448e+006**	**2,930e+006**	**4,693e+006**
805		0,003	1,4	1,004e+006	9,106e+005	1,291e+006
**1312**	**Adenylate kinase isoenzyme**	**0,003**	**1,5**	**7,847e+005**	**8,606e+005**	**1,195e+006**
1089		0,004	1,3	2,022e+006	1,853e+006	2,467e+006
604		0,005	1,6	7,856e+006	9,104e+006	1,282e+007
**1681**	**Creatine kinase M-type**	**0,005**	**1,4**	**6,657e+006**	**6,574e+006**	**8,885e+006**
**1733**	**Creatine kinase M-type**	**0,005**	**1,4**	**2,181e+006**	**2,319e+006**	**3,022e+006**
**1211**	**Carbonic anhydrase 1**	**0,005**	**1,4**	**3,775e+006**	**4,042e+006**	**5,285e+006**
597		0,006	1,5	2,531e+006	1,843e+006	2,762e+006
1078		0,008	1,4	1,588e+007	1,565e+007	2,194e+007
591		0,008	1,4	3,636e+006	2,684e+006	3,530e+006
796		0,008	1,5	4,132e+005	5,257e+005	6,248e+005
**1716**	**Creatine kinase M-type**	**0,011**	**1,6**	**1,016e+006**	**9,177e+005**	**1,426e+006**
914		0,011	1,5	2,303e+006	2,615e+006	3,388e+006
**509**	**Creatine kinase M-type**	**0,013**	**1,7**	**1,608e+006**	**1,269e+006**	**2,186e+006**
1074		0,013	1,3	2,241e+007	1,920e+007	2,556e+007
1172		0,014	1,3	4,249e+006	3,593e+006	4,721e+006
**1042**	**Creatine kinase M-type**	**0,014**	**1,6**	**2,605e+006**	**2,488e+006**	**3,970e+006**
578		0,016	1,5	1,124e+006	7,283e+005	1,081e+006
374		0,016	1,8	1,246e+006	9,526e+005	1,695e+006
1156		0,017	1,3	9,904e+005	1,152e+006	1,329e+006
1094		0,018	1,8	8,947e+005	1,066e+006	1,641e+006

Spot	Identification	Anova (p)	Fold	Average Normalised Volumes		
				18ºC	24ºC	30ºC
**1529**	**Neuroendocrine convertase 1**	**0,018**	**1,6**	**4,067e+006**	**3,746e+006**	**5,877e+006**
1228		0,020	1,3	3,820e+006	3,035e+006	3,180e+006
1666		0,020	1,9	1,757e+005	1,910e+005	3,384e+005
**1295**	**Adenylate kinase isoenzyme 1**	**0,021**	**1,6**	**1,108e+006**	**1,429e+006**	**1,805e+006**
13		0,022	1,8	1,699e+006	1,229e+006	9,353e+005
1072		0,022	1,4	7,265e+006	6,155e+006	8,706e+006
732		0,022	1,3	8,047e+005	9,315e+005	1,053e+006
385		0,022	1,6	3,797e+006	2,390e+006	3,318e+006
**1720**	**Creatine kinase M-type**	**0,023**	**1,1**	**4,170e+007**	**4,156e+007**	**3,692e+007**
**1246**	**Triosephosphate isomerase B**	**0,024**	**1,3**	**1,593e+007**	**2,021e+007**	**2,033+007**
544		0,025	1,8	1,730e+006	2,231e+006	3,074e+006
**1674**	**Actin, alpha cardiac muscle 2**	**0,026**	**1,6**	**1,660e+005**	**1,942e+005**	**2,737e+005**
1336		0,028	1,4	1,295e+007	1,018e+007	1,420e+007
**857**	**Alpha-enolase**	**0,029**	**1,6**	**1,028e+006**	**1,013e+006**	**1,666e+006**
1237		0,029	1,7	5,212e+005	4,214e+005	7,025e+005
**1143**	**Creatine kinase, testis isozyme**	**0,031**	**1,5**	**1,260e+006**	**1,566e+006**	**1,865e+006**
**897**	**Eukaryotic initiation factor 4A-II**	**0,034**	**1,4**	**1,438e+006**	**1,068e+006**	**1,478e+006**
**851**	**Alpha-enolase**	**0,046**	**1,3**	**2,173e+006**	**2,253e+006**	**2,872e+006**
1212		0,047	1,3	1,050e+006	9,366e+005	1,175e+006
1102		0,047	1,5	1,323e+006	1,377e+006	1,924e+006
887		0,048	1,6	2,532e+005	2,857e+005	3,958e+005
**428**	**Glycogen phosphorylase, brain form**	**0,049**	**2,0**	**1,725e+006**	**8,464e+005**	**1,418e+006**

Table S3. Masses and sequences of peptides obtained for each spot at **a)**14 days of exposure, **b)** 21 days of exposure.

a) 14 days of exposure

**SPOT nº**	Protein Name	Species	Accession Number	Protein MW	Protein PI	Peptide Count	Protein Score	Protein Score C.I. %
**859**	Rab GDP dissociation inhibitor beta	*Canis familiaris*	GDIB_CANFA	50289.73828	6.11	2	111	100
	Calculated Mass	Observed Mass	Match Error PPM	Start Sequence Position	End Sequence Position	Sequence	Ion Score	Ion Score C.I. %
	1993.947	1993.8823	-32	330	348	SDIYVCMISSAHNVAAQGK		
	2141.1064	2141.186	37	222	240	SPYLYPLYGLGELPQGFAR	99.63999939	100
	2141.1064	2141.186	37	222	240	SPYLYPLYGLGELPQGFAR		

	Protein Name	Species	Accession Number	Protein MW	Protein PI	Peptide Count	Protein Score	Protein Score C.I. %
**1529**	Neuroendocrine convertase 1	*Homo sapiens*	NEC1_HUMAN	84099.28125	5.66	5	64	96.6738544
	Calculated Mass	Observed Mass	Match Error PPM	Start Sequence Position	End Sequence Position	Sequence	Ion Score	Ion Score C.I. %
	1994.9454	1994.8646	-40	90	105	LSDDDRVIWAEQQYEK		
	1994.9454	1994.8646	-40	90	105	LSDDDRVIWAEQQYEK		
	2268.0925	2268.0195	-32	618	637	RGVEKMVDPGEEQPTQENPK		
	2788.3884	2788.3342	-19	4	28	RAWSLQCTAFVLFCAWCALNSAKAK		
	2788.3884	2788.3342	-19	5	29	AWSLQCTAFVLFCAWCALNSAKAKR		

	Protein Name	Species	Accession Number	Protein MW	Protein PI	Peptide Count	Protein Score	Protein Score C.I. %
**851**	Alpha-enolase	*Xenopus laevis*	ENOA_XENLA	47474	5.92	12	159	100
	Calculated Mass	Observed Mass	Match Error PPM	Start Sequence Position	End Sequence Position	Sequence	Ion Score	Ion Score C.I. %
	1143.6157	1143.6514	31	184	193	IGAEVYHNLK		
	1541.7642	1541.7538	-7	359	372	LAQSNGWGVMVSHR		
	1554.7072	1554.729	14	257	269	YDLDFKSPDDPSR		
	1804.944	1804.8773	-37	33	50	AAVPSGASTGIYEALELR	97.34999847	100
	1804.944	1804.8773	-37	33	50	AAVPSGASTGIYEALELR		
	1851.9191	1851.851	-37	90	105	IDKLMIEMDGTENKSK		
	1960.9247	1960.9109	-7	203	221	DATNVGDEGGFAPNILENK		
	2277.1357	2277.0425	-41	33	54	AAVPSGASTGIYEALELRDNDK		
	2501.3721	2501.358	-6	307	330	FTAASGIQVVGDDLTVTNPKRIAK		
	2508.2439	2508.2074	-15	68	89	YVNEFLGPALCTQNLNVVEQEK		
	2566.2419	2566.2776	14	198	221	EKYGKDATNVGDEGGFAPNILENK		
	2757.394	2757.35	-16	203	228	DATNVGDEGGFAPNILENKEALELLK		
	3021.5903	3021.537	-18	133	162	HIADLAGNPEVILPVPAFNVINGGSHAGNK		

	Protein Name	Species	Accession Number	Protein MW	Protein PI	Peptide Count	Protein Score	Protein Score C.I. %
**1733**	Creatine kinase M-type	*Bos taurus*	KCRM_BOVIN	42962	6.63	7	132	100
	Calculated Mass	Observed Mass	Match Error PPM	Start Sequence Position	End Sequence Position	Sequence	Ion Score	Ion Score C.I. %
	1507.7023	1507.7299	18	117	130	GGDDLDPNYVLSSR		
	1643.8176	1643.8443	16	224	236	SFLVWVNEEDHLR	59.79000092	99.982
	1643.8176	1643.8443	16	224	236	SFLVWVNEEDHLR		

1785.9592	1785.9634	2	342	358	LGSSEVEQVQLVVDGVK		
1800.9524	1800.9608	5	366	381	KLEKGQSIDDMIPAQK		
1994.9414	1994.9568	8	321	341	GTGGVDTAAVGSVFDVSNADR	32.34999847	90.103
1994.9414	1994.9568	8	321	341	GTGGVDTAAVGSVFDVSNADR		
2151.0425	2150.9629	-37	320	341	RGTGGVDTAAVGSVFDVSNADR		
3761.8826	3761.7926	-24	321	358	GTGGVDTAAVGSVFDVSNADRLGSSEVEQVQLVVDGVK		

	Protein Name	Species	Accession Number	Protein MW	Protein PI	Peptide Count	Protein Score	Protein Score C.I. %
**1246**	Triosephosphate isomerase B	*Danio rerio*	TPISB_DANRE	26810.80078	6.45	8	138	100

Calculated Mass	Observed Mass	Match Error PPM	Start Sequence Position	End Sequence Position	Sequence	Ion Score	Ion Score C.I. %
1082.5781	1082.5544	-22	5	13	KFFVGGNWK		
1096.5898	1096.574	-14	90	98	WVILGHSER	84.80999756	100
1096.5898	1096.574	-14	90	98	WVILGHSER		
1252.6909	1252.6694	-17	90	99	WVILGHSERR		
1458.7223	1458.6913	-21	100	112	HVFGESDELIGQK		
1602.889	1602.835	-34	160	174	VVLAYEPVWAIGTGK		
1614.8234	1614.8153	-5	99	112	RHVFGESDELIGQK		
2034.0865	2034.0222	-32	131	148	LDEREAGITEKVVFAQTK		
2432.3071	2432.2161	-37	225	247	DLDGFLVGGASLKPEFIDIINAK		

	Protein Name	Species	Accession Number	Protein MW	Protein PI	Peptide Count	Protein Score	Protein Score C.I. %
**1042**	Creatine kinase M-type	*Homo sapiens*	KCRM_HUMAN	43073.89844	6.77	5	78.90000153	99.89236808

Calculated Mass	Observed Mass	Match Error PPM	Start Sequence Position	End Sequence Position	Sequence	Ion Score	Ion Score C.I. %
1267.6542	1267.6226	-25	97	107	HGGYKPTDKHK		
1643.8176	1643.7942	-14	224	236	SFLVWVNEEDHLR	55.06000137	99.958
1643.8176	1643.7942	-14	224	236	SFLVWVNEEDHLR		
2151.0425	2151.0168	-12	320	341	RGTGGVDTAAVGSVFDVSNADR		
3348.5933	3348.4941	-30	210	236	DWPDARGIWHNDNKSFLVWVNEEDHLR		
3808.9116	3808.7048	-54	183	215	EQQQLIDDHFLFDKPVSPLLLASGMARDWPDAR		

	Protein Name	Species	Accession Number	Protein MW	Protein PI	Peptide Count	Protein Score	Protein Score C.I. %
**1720**	Creatine kinase M-type	*Homo sapiens*	KCRM_HUMAN	43073.89844	6.77	10	213	100

Calculated Mass	Observed Mass	Match Error PPM	Start Sequence Position	End Sequence Position	Sequence	Ion Score	Ion Score C.I. %
1002.5002	1002.4893	-11	97	105	HGGYKPTDK		
1037.5197	1037.4858	-33	243	251	GGNMKEVFR		
1507.7023	1507.6552	-31	117	130	GGDDLDPNYVLSSR		
1553.8396	1553.7616	-50	253	265	FCVGLQKIEEIFK		
1643.8176	1643.8132	-3	224	236	SFLVWVNEEDHLR	112	100
1643.8176	1643.8132	-3	224	236	SFLVWVNEEDHLR		
1724.8378	1724.8068	-18	12	25	LNYKPEEEYPDLSK		
1785.9592	1785.9015	-32	342	358	LGSSEVEQVQLVVDGVK		
1994.9414	1994.9055	-18	321	341	GTGGVDTAAVGSVFDVSNADR	68.12000275	99.999
1994.9414	1994.9055	-18	321	341	GTGGVDTAAVGSVFDVSNADR		
2151.0425	2150.9807	-29	320	341	RGTGGVDTAAVGSVFDVSNADR		
3761.8826	3761.7659	-31	321	358	GTGGVDTAAVGSVFDVSNADRLGSSEVEQVQLVVDGVK		

	Protein Name	Species	Accession Number	Protein MW	Protein PI	Peptide Count	Protein Score	Protein Score C.I. %
**1716**	Creatine kinase M-type	*Sus scrofa*	KCRM_PIG	43031.85156	6.61	14	78.09999847	99.87059787
	Calculated Mass	Observed Mass	Match Error PPM	Start Sequence Position	End Sequence Position	Sequence	Ion Score	Ion Score C.I. %
	1002.5002	1002.4925	-8	97	105	HGGYKPTDK		
	1037.5197	1037.5169	-3	243	251	GGNMKEVFR		
	1045.4884	1045.5146	25	1	9	MPFGNTHNK		
	1193.6017	1193.5699	-27	237	247	VISMEKGGNMK		
	1205.6062	1205.5931	-11	2	11	PFGNTHNKYK		
	1302.6359	1302.6357	0	370	381	GQSIDDMIPAQK		
	1507.7023	1507.7317	19	117	130	GGDDLDPNYVLSSR		
	1628.8074	1628.7775	-18	139	152	GYTLPPHCSRGERR		
	1643.8176	1643.848	18	224	236	SFLVWVNEEDHLR		
	1785.9592	1785.9655	4	342	358	LGSSEVEQVQLVVDGVK		
	1838.937	1838.8656	-39	1	15	MPFGNTHNKYKLNFK		
	1994.9414	1994.8635	-39	321	341	GTGGVDTAAVGSVFDVSNADR	39.20000076	99.38
	1994.9414	1994.8635	-39	321	341	GTGGVDTAAVGSVFDVSNADR		
	2151.0425	2150.9619	-37	320	341	RGTGGVDTAAVGSVFDVSNADR		
	3348.5933	3348.5507	-13	210	236	DWPDARGIWHNDNKSFLVWVNEEDHLR		

	Protein Name	Species	Accession Number	Protein MW	Protein PI	Peptide Count	Protein Score	Protein Score C.I. %
**1670**	Heat shock 70 kDa protein 1	*Oryzias latipes*	HSP71_ORYLA	70307.00781	5.47	16	370	100
	Calculated Mass	Observed Mass	Match Error PPM	Start Sequence Position	End Sequence Position	Sequence	Ion Score	Ion Score C.I. %
	1017.5687	1017.532	-36	503	511	ITITNDKGR		

1109.5739	1109.576	2	351	359	LLQDFFNGR		
1193.5645	1193.5656	1	553	563	SSAQDDSLKDK		
1228.6281	1228.5903	-31	28	38	VEIIANDQGNR		
1235.6631	1235.6992	29	542	552	NSLESLAFNLK		
1434.7046	1434.7246	14	79	90	RFDEPVVQADMK		
1487.7013	1487.6521	-33	39	51	TTPSYVAFTDTER	81.08999634	100
1487.7013	1487.6521	-33	39	51	TTPSYVAFTDTER		
1501.7605	1501.7688	6	529	541	AEDEQQRDKIAAK		
1628.9039	1628.8523	-32	598	611	QKELEKVCNPIISK		
1659.8951	1659.8319	-38	174	189	IINEPTAAAIAYGLDK		
1675.7307	1675.6835	-28	223	238	ATAGDTHLGGEDFDNR	91.13999939	100
1675.7307	1675.6835	-28	223	238	ATAGDTHLGGEDFDNR		
1845.0116	1844.99271	-10	174	191	IINEPTAAAIAYGLDKGK		
1880.9501	1880.8909	-31	141	157	VSNAVITVPAYFNDSQR	149.2400055	100
1880.9501	1880.8909	-31	141	157	VSNAVITVPAYFNDSQR		
2286.2087	2286.15	-26	52	73	LIGDAAKNQVALNPSNTVFDAK		
2309.1885	2309.171	-8	141	161	VSNAVITVPAYFNDSQRQATK		
2981.3364	2981.3696	11	612	639	LYQGGMPSGSCREQARADSQGPTIEEVD		

	Protein Name	Species	Accession Number	Protein MW	Protein PI	Peptide Count	Protein Score	Protein Score C.I. %
**1668**	Heat shock cognate 71 kDa protein	*Cricetulus griseus*	HSP7C_CRIGR	70761.11719	5.24	3	87.59999847	99.98548084

Calculated Mass	Observed Mass	Match Error PPM	Start Sequence Position	End Sequence Position	Sequence	Ion Score	Ion Score C.I. %
1480.7543	1480.7427	-8	300	311	ARFEELNADLFR		
2774.3267	2774.2542	-26	424	447	QTQTFTTYSDNQPGVLIQVYEGER	72.94999695	99.999
2774.3267	2774.2542	-26	424	447	QTQTFTTYSDNQPGVLIQVYEGER		

	2956.5051	2956.445	-20	129	155	EIAEAYLGKTVTNAVVTVPAYFNDSQR		

	Protein Name	Species	Accession Number	Protein MW	Protein PI	Peptide Count	Protein Score	Protein Score C.I. %
**1312**	Adenylate kinase isoenzyme 1	*Cyprinus carpio*	KAD1_CYPCA	21475.33984	6.64	2	90.40000153	99.99238024
	Calculated Mass	Observed Mass	Match Error PPM	Start Sequence Position	End Sequence Position	Sequence	Ion Score	Ion Score C.I. %
	1053.5364	1053.573	34	89	97	GYLIDGYPR		
	1481.7383	1481.682	-38	32	44	YGYTHLSSGDLLR	81.72000122	100
	1481.7383	1481.682	-38	32	44	YGYTHLSSGDLLR		

	Protein Name	Species	Accession Number	Protein MW	Protein PI	Peptide Count	Protein Score	Protein Score C.I. %
**1681**	Creatine kinase M-type	*Canis familiaris*	KCRM_CANFA	43125.89844	6.77	5	126	100
	Calculated Mass	Observed Mass	Match Error PPM	Start Sequence Position	End Sequence Position	Sequence	Ion Score	Ion Score C.I. %
	1037.5197	1037.5306	11	243	251	GGNMKEVFR		
	1507.7023	1507.6902	-8	117	130	GGDDLDPNYVLSSR		
	1657.8333	1657.8374	2	224	236	TFLVWVNEEDHLR	58.52999878	99.972
	1657.8333	1657.8374	2	224	236	TFLVWVNEEDHLR		
	1785.9592	1785.967	4	342	358	LGSSEVEQVQLVVDGVK	48.02000046	99.682
	1785.9592	1785.967	4	342	358	LGSSEVEQVQLVVDGVK		

	Protein Name	Species	Accession Number	Protein MW	Protein PI	Peptide Count	Protein Score	Protein Score C.I. %

**1295**	Adenylate kinase isoenzyme 1	*Cyprinus carpio*	KAD1_CYPCA	21475.33984	6.64	4	88.5	99.98819839
	Calculated Mass	Observed Mass	Match Error PPM	Start Sequence Position	End Sequence Position	Sequence	Ion Score	Ion Score C.I. %
	1053.5364	1053.5208	-15	89	97	GYLIDGYPR		
	1350.6899	1350.684	-4	98	108	EVKQGEEFEKK		
	1481.7383	1481.6904	-32	32	44	YGYTHLSSGDLLR	79.58000183	100
	1481.7383	1481.6904	-32	32	44	YGYTHLSSGDLLR		
	1837.9807	1837.91957	-33	156	171	ATEPVIAYYETRGIVR		

	Protein Name	Species	Accession Number	Protein MW	Protein PI	Peptide Count	Protein Score	Protein Score C.I. %
**857**	Alpha-enolase	*Pongo abelii*	ENOA_PONAB	47167.35938	7.57	10	108	100
	Calculated Mass	Observed Mass	Match Error PPM	Start Sequence Position	End Sequence Position	Sequence	Ion Score	Ion Score C.I. %
	1143.6157	1143.614	-1	184	193	IGAEVYHNLK		
	1434.7444	1434.7045	-28	331	343	AVNEKSCNCLLLK		
	1554.7072	1554.6644	-28	257	269	YDLDFKSPDDPSR		
	1804.944	1804.8762	-38	33	50	AAVPSGASTGIYEALELR	58.84000015	99.984
	1804.944	1804.8762	-38	33	50	AAVPSGASTGIYEALELR		
	1826.8556	1826.8005	-30	254	269	SGKYDLDFKSPDDPSR		
	1901.0055	1900.942	-33	270	285	YISPDQLADLYKSFIK		
	1960.9247	1960.9348	5	203	221	DATNVGDEGGFAPNILENK		
	2277.1357	2277.193	25	33	54	AAVPSGASTGIYEALELRDNDK		
	2501.3721	2501.2555	-47	307	330	FTASAGIQVVGDDLTVTNPKRIAK		
	2743.3784	2743.317	-22	203	228	DATNVGDEGGFAPNILENKEGLELLK		

	Protein Name	Species	Accession Number	Protein MW	Protein PI	Peptide Count	Protein Score	Protein Score C.I. %
**1674**	Actin, alpha cardiac muscle 2	*Xenopus tropicalis*	ACT2_XENTR	42005.89844	5.23	9	115	100
	Calculated Mass	Observed Mass	Match Error PPM	Start Sequence Position	End Sequence Position	Sequence	Ion Score	Ion Score C.I. %
	1130.5476	1130.5159	-28	199	208	GYSFVTTAER		
	1161.6184	1161.5804	-33	318	328	EITALAPSTMK		
	1198.7054	1198.745	33	31	41	AVFPSIVGRPR		
	1198.7054	1198.745	33	31	41	AVFPSIVGRPR		
	1500.7078	1500.66	-32	362	374	QEYDEAGPSIVHR		
	1515.7491	1515.6948	-36	87	97	IWHHTFYNELR		
	1628.8027	1628.769	-21	362	375	QEYDEAGPSIVHRK		
	1790.892	1790.8229	-39	241	256	SYELPDGQVITIGNER	56.70999908	99.975
	1790.892	1790.8229	-39	241	256	SYELPDGQVITIGNER		
	1956.0437	1955.972	-37	98	115	VAPEEHPTLLTEAPLNPK		
	3793.9561	3793.89	-17	87	118	IWHHTFYNELRVAPEEHPTLLTEAPLNPKANR		

	Protein Name	Species	Accession Number	Protein MW	Protein PI	Peptide Count	Protein Score	Protein Score C.I. %
**428**	Glycogen phosphorylase, brain form	*Ovis aries*	PYGB_SHEEP	96253.39063	6.57	10	93.90000153	99.99659638
	Calculated Mass	Observed Mass	Match Error PPM	Start Sequence Position	End Sequence Position	Sequence	Ion Score	Ion Score C.I. %
	1053.5728	1053.5591	-13	643	650	VIFLENYR		
	1145.5626	1145.5304	-28	162	170	YEFGIFNQK		
	1355.743	1355.7541	8	400	410	HLDIIYAINQR		
	1442.6951	1442.7335	27	279	290	VLYPNDNFFEGK		

1478.7737	1478.7134	-41	508	520	IGEDFLTDLSQLK		
1829.8817	1829.8486	-18	171	185	IVNGWQVEEADDWLR	60.40999985	99.985
1829.8817	1829.8486	-18	171	185	IVNGWQVEEADDWLR		
1840.9227	1840.8696	-29	279	293	VLYPNDNFFEGKELR		
1845.0051	1844.9572	-25	593	609	KDPTQAFVPRTVMIGGK		
2512.3606	2512.2731	-35	571	590	IHEYKRQLLNCLHVVTLYNR		
2691.2871	2691.2356	-19	774	796	VFADYEAYVACQAQVDQLYRNPK		

	Protein Name	Species	Accession Number	Protein MW	Protein PI	Peptide Count	Protein Score	Protein Score C.I. %
**897**	Eukaryotic initiation factor 4A-II	*Macaca fascicularis*	IF4A2_MACFA	46387.78906	5.4	12	146	100

Calculated Mass	Observed Mass	Match Error PPM	Start Sequence Position	End Sequence Position	Sequence	Ion Score	Ion Score C.I. %
1065.551	1065.543	-8	314	321	DVIMREFR		
1114.6831	1114.6416	-37	327	336	VLITTDLLAR		
1157.6174	1157.6372	17	356	364	ENYIHRIGR		
1174.658	1174.6939	31	285	293	RKVDWLTEK		
1198.6791	1198.645	-28	102	112	ETQALVLAPTR		
1394.691	1394.642	-35	71	84	GYDVIAQAQSGTGK		
1483.6919	1483.693	1	180	192	MFVLDEADGMLSR		
1539.7697	1539.732	-24	314	326	DVIMREFRSGSSR		
1638.8784	1638.831	-29	164	176	VFDMLNRRYLSPK		
1827.9388	1827.9525	7	48	63	GIYAYGFEKPSAIQQR	65.13999939	99.997
1827.9388	1827.9525	7	48	63	GIYAYGFEKPSAIQQR		
2144.1345	2144.1312	-2	337	355	GIDVQQVSLVINYDLPTNR	47.11000061	99.841
2144.1345	2144.1312	-2	337	355	GIDVQQVSLVINYDLPTNR		
2499.2773	2499.17641	-40	172	192	YLSPKWIKMFVLDEADGMLSR		

	Protein Name	Species	Accession Number	Protein MW	Protein PI	Peptide Count	Protein Score	Protein Score C.I. %
**509**	Creatine kinase M-type	*Homo sapiens*	KCRM_HUMAN	43073.89844	6.77	8	156	100
	Calculated Mass	Observed Mass	Match Error PPM	Start Sequence Position	End Sequence Position	Sequence	Ion Score	Ion Score C.I. %
	1189.6113	1189.5642	-40	2	11	PFGNTHNKFK		
	1507.7023	1507.7218	13	117	130	GGDDLDPNYVLSSR		
	1643.8176	1643.7619	-34	224	236	SFLVWVNEEDHLR	71.16999817	100
	1643.8176	1643.7619	-34	224	236	SFLVWVNEEDHLR		
	1785.9592	1785.9608	1	342	358	LGSSEVEQVQLVVDGVK		
	1994.9414	1994.955	7	321	341	GTGGVDTAAVGSVFDVSNADR	47.75	99.834
	1994.9414	1994.955	7	321	341	GTGGVDTAAVGSVFDVSNADR		
	2151.0425	2150.9948	-22	320	341	RGTGGVDTAAVGSVFDVSNADR		
	3348.5933	3348.4729	-36	210	236	DWPDARGIWHNDNKSFLVWVNEEDHLR		
	3808.9116	3808.866	-12	183	215	EQQQLIDDHFLFDKPVSPLLLASGMARDWPDAR		

	Protein Name	Species	Accession Number	Protein MW	Protein PI	Peptide Count	Protein Score	Protein Score C.I. %
**1143**	Creatine kinase, testis isozyme	*Oncorhynchus mykiss*	KCRT_ONCMY	42976.76172	6.2	3	57.5	85.14265336
	Calculated Mass	Observed Mass	Match Error PPM	Start Sequence Position	End Sequence Position	Sequence	Ion Score	Ion Score C.I. %
	1638.8633	1638.8318	-19	35	47	VLTQDMYTKLRDR		
	1657.8333	1657.8588	15	226	238	TFLVWVNEEDHLR	45.29000092	99.424
	1657.8333	1657.8588	15	226	238	TFLVWVNEEDHLR		
	2691.4346	2691.408	-10	344	367	LGFSEVELVQMVVDGVKLLVEMEK		

	Protein Name	Species	Accession Number	Protein MW	Protein PI	Peptide Count	Protein Score	Protein Score C.I. %
**1211**	Carbonic anhydrase 1	*Chionodraco hamatus*	CAH1_CHIHA	28324.91992	5.58	2	106	100
	Calculated Mass	Observed Mass	Match Error PPM	Start Sequence Position	End Sequence Position	Sequence	Ion Score	Ion Score C.I. %
	1607.8329	1607.8223	-7	113	125	YPAELHLVHWNTK	94.48000336	100
	1607.8329	1607.8223	-7	113	125	YPAELHLVHWNTK		
	2811.3274	2811.2783	-17	1	26	AHAWGYGPTDGPDKWVSNFPIADGPR		

	Protein Name	Species	Accession Number	Protein MW	Protein PI	Peptide Count	Protein Score	Protein Score C.I. %
**1669**	Heat shock 70 kDa protein 1	*Oryzias latipes*	HSP71_ORYLA	70307.00781	5.47	11	149	100
	Calculated Mass	Observed Mass	Match Error PPM	Start Sequence Position	End Sequence Position	Sequence	Ion Score	Ion Score C.I. %
	1109.5739	1109.577	3	351	359	LLQDFFNGR	49.00999832	99.823
	1109.5739	1109.577	3	351	359	LLQDFFNGR		
	1435.7151	1435.7135	-1	239	249	MVNHFVEEFKR		
	1487.7013	1487.7169	10	39	51	TTPSYVAFTDTER		
	1628.9039	1628.914	6	598	611	QKELEKVCNPIISK		
	1659.8951	1659.8582	-22	174	189	IINEPTAAAIAYGLDK		
	1675.7307	1675.7623	19	223	238	ATAGDTHLGGEDFDNR		
	1880.9501	1880.9473	-1	141	157	VSNAVITVPAYFNDSQR	57.63000107	99.976
	1880.9501	1880.9473	-1	141	157	VSNAVITVPAYFNDSQR		
	2309.1885	2309.1375	-22	141	161	VSNAVITVPAYFNDSQRQATK		
	2801.4429	2801.4193	-8	105	128	IQVDYKGENKTFFPEEISSMVLVK		
	2936.3267	2936.4236	33	223	248	ATAGDTHLGGEDFDNRMVNHFVEEFK		

2981.3364	2981.4426	36	612	639	LYQGGMPSGSCREQARADSQGPTIEEVD

b) 21 days of exposure

SPOT nº	Protein Name	Species	Accession Number	Protein MW	Protein PI	Peptide Count	Protein Score	Protein Score C.I. %
**1781**	Heat shock 70 kDa protein 1	*Oryzias latipes*	HSP71_ORYLA	70307.00781	5.47	9	171	100
	Calculated Mass	Observed Mass	Match Error PPM	Start Sequence Position	End Sequence Position	Sequence	Ion Score	Ion Score C.I. %
	1109.5739	1109.5664	-7	351	359	LLQDFFNGR	40.29999924	97.175
	1109.5739	1109.5664	-7	351	359	LLQDFFNGR		
	1487.7013	1487.7029	1	39	51	TTPSYVAFTDTER		
	1501.7605	1501.7949	23	529	541	AEDEQQRDKIAAK		
	1628.9039	1628.9182	9	598	611	QKELEKVCNPIISK		
	1659.8951	1659.87	-15	174	189	IINEPTAAAIAYGLDK		
	1675.7307	1675.7318	1	223	238	ATAGDTHLGGEDFDNR		
	1845.0116	1844.9695	-23	174	191	IINEPTAAAIAYGLDKGK		
	1880.9501	1880.9434	-4	141	157	VSNAVITVPAYFNDSQR	80.33000183	100
	1880.9501	1880.9434	-4	141	157	VSNAVITVPAYFNDSQR		
	2309.1885	2309.1162	-31	141	161	VSNAVITVPAYFNDSQRQATK		

	Protein Name	Species	Accession Number	Protein MW	Protein PI	Peptide Count	Protein Score	Protein Score C.I. %
**1389**	Adenylate kinase isoenzyme 1	*Cyprinus carpio*	KAD1_CYPCA	21475.33984	6.64	2	91.80000305	99.99447998

	Calculated Mass	Observed Mass	Match Error PPM	Start Sequence Position	End Sequence Position	Sequence	Ion Score	Ion Score C.I. %
	1053.5364	1053.5267	-9	89	97	GYLIDGYPR		
	1481.7383	1481.7466	6	32	44	YGYTHLSSGDLLR	70.86000061	99.992
	1481.7383	1481.7466	6	32	44	YGYTHLSSGDLLR		

	Protein Name	Species	Accession Number	Protein MW	Protein PI	Peptide Count	Protein Score	Protein Score C.I. %
**475**	Heat shock cognate 71 kDa protein	*Ictalurus punctatus*	HSP7C_ICTPU	71296.14063	5.19	12	148	100
	Calculated Mass	Observed Mass	Match Error PPM	Start Sequence Position	End Sequence Position	Sequence	Ion Score	Ion Score C.I. %
	1197.6626	1197.6881	21	459	469	FELTGIPPAPR		
	1215.6692	1215.6469	-18	160	171	DAGTISGLNVLR		
	1252.6605	1252.6339	-21	127	137	MKEIAEAYLGK		
	1473.6857	1473.6835	-1	37	49	TTPSYVAFTDSER		
	1480.7543	1480.7748	14	300	311	ARFEELNADLFR		
	1691.7256	1691.7026	-14	221	236	STAGDTHLGGEDFDNR		
	1787.9901	1787.96	-17	172	188	IINEPTAAAIAYGLDKK		
	1807.9775	1807.8646	-62	343	357	IPKMEKLLQDFFNGK		
	1963.9371	1964.00272	33	78	93	FEDSVVQADMKHWPFK		
	2009.0087	2009.0255	8	138	155	SINNAVITVPAYFNDSQR		
	2774.3267	2774.3042	-8	424	447	QTQTFTTYSDNQPGVLIQVYEGER	52.68999863	99.809
	2774.3267	2774.3042	-8	424	447	QTQTFTTYSDNQPGVLIQVYEGER		
	2776.3635	2776.337	-10	358	384	ELNKSINPDEAVAYGAAVQAESSLGDK		

	Protein Name	Species	Accession Number	Protein MW	Protein PI	Peptide Count	Protein Score	Protein Score C.I. %

**1331**	Triosephosphate isomerase (Fragments)	*Mesocricetus auratus*	TPIS_MESAU	20294.36914	5.49	1	83.5	99.96268003
	Calculated Mass	Observed Mass	Match Error PPM	Start Sequence Position	End Sequence Position	Sequence	Ion Score	Ion Score C.I. %
	1602.889	1602.8638	-16	160	174	VVLAYEPVWAIGTGK	50.81999969	99.527
	1602.889	1602.8638	-16	160	174	VVLAYEPVWAIGTGK		

	Protein Name	Species	Accession Number	Protein MW	Protein PI	Peptide Count	Protein Score	Protein Score C.I. %
**1017**	Glyceraldehyde-3-phosphate dehydrogenase	*Danio rerio*	G3P_DANRE	35761.30078	8.2	3	62.40000153	95.19225856
	Calculated Mass	Observed Mass	Match Error PPM	Start Sequence Position	End Sequence Position	Sequence	Ion Score	Ion Score C.I. %
	1495.8479	1495.8491	1	233	246	VPTPNVSVVDLTVR		
	1763.8024	1763.8037	1	308	321	LVTWYDNEFGYSNR	40.22999954	96.548
	1763.8024	1763.8037	1	308	321	LVTWYDNEFGYSNR		

	Protein Name	Species	Accession Number	Protein MW	Protein PI	Peptide Count	Protein Score	Protein Score C.I. %
**989**	Glyceraldehyde-3-phosphate dehydrogenase (Fragment)	*Meleagris gallopavo*	G3P_MELGA	24836.68945	7.22	3	123	100
	Calculated Mass	Observed Mass	Match Error PPM	Start Sequence Position	End Sequence Position	Sequence	Ion Score	Ion Score C.I. %

	Calculated Mass	Observed Mass	Match Error PPM	Start Sequence Position	End Sequence Position	Sequence	Ion Score	Ion Score C.I. %
	1697.8929	1697.879	-8	107	124	DGRGAAQNIIPASTGAAK		
	1749.7867	1749.8069	12	219	232	LVSWYDNEFGYSNR	98.09999847	100
	1749.7867	1749.8069	12	219	232	LVSWYDNEFGYSNR		
	2257.1467	2257.0627	-37	137	157	LTGMAFHVPTPNVSVVDLTCR		

	Protein Name	Species	Accession Number	Protein MW	Protein PI	Peptide Count	Protein Score	Protein Score C.I. %
**1086**	Sterile alpha motif domain-containing protein 9-like	*Homo sapiens*	SAM9L_HUMAN	184415.3906	8.25	8	67.40000153	98.47965866
	Calculated Mass	Observed Mass	Match Error PPM	Start Sequence Position	End Sequence Position	Sequence	Ion Score	Ion Score C.I. %
	1127.6095	1127.6201	9	1471	1480	QASTLFYLGK		
	1127.6095	1127.6201	9	1471	1480	QASTLFYLGK		
	1551.9945	1551.8969	-63	1399	1411	LIQPLTTLKKQLR		
	1600.917	1600.943	16	1546	1560	IPVISVYSGPLRSGR	37.29000092	90
	1600.917	1600.943	16	1546	1560	IPVISVYSGPLRSGR		
	1842.0959	1841.9996	-52	1544	1560	IKIPVISVYSGPLRSGR		
	1874.9792	1874.9623	-9	1220	1235	ENELSKKHMVQFLSGK		
	1874.9792	1874.9623	-9	1220	1235	ENELSKKHMVQFLSGK		
	1954.8777	1954.938	31	1169	1184	QTDSKNYETENWSPQK		
	1954.8777	1954.938	31	1169	1184	QTDSKNYETENWSPQK		
	2783.3845	2783.3286	-20	894	917	SNFDETYIENVVRNILKGQDVDSK		
	2784.511	2784.4167	-34	1374	1398	KPMTNEKQNSILANIILSCLKPNSK		

	Protein Name	Species	Accession Number	Protein MW	Protein PI	Peptide Count	Protein Score	Protein Score C.I. %
**999**	Glyceraldehyde-3-	*Danio rerio*	G3P_DANRE	35761.30078	8.2	2	163	100

	phosphate dehydrogenase							
	Calculated Mass	Observed Mass	Match Error PPM	Start Sequence Position	End Sequence Position	Sequence	Ion Score	Ion Score C.I. %
	1495.8479	1495.8875	26	233	246	VPTPNVSVVDLTVR	68.76000214	99.993
	1495.8479	1495.8875	26	233	246	VPTPNVSVVDLTVR		
	1763.8024	1763.84	21	308	321	LVTWYDNEFGYSNR	79.01999664	100
	1763.8024	1763.84	21	308	321	LVTWYDNEFGYSNR		

	Protein Name	Species	Accession Number	Protein MW	Protein PI	Peptide Count	Protein Score	Protein Score C.I. %
**1007**	Glyceraldehyde-3-phosphate dehydrogenase	*Danio rerio*	G3P_DANRE	35761.30078	8.2	2	146	100
	Calculated Mass	Observed Mass	Match Error PPM	Start Sequence Position	End Sequence Position	Sequence	Ion Score	Ion Score C.I. %
	1495.8479	1495.851	2	233	246	VPTPNVSVVDLTVR	47.68999863	99.023
	1495.8479	1495.851	2	233	246	VPTPNVSVVDLTVR		
	1763.8024	1763.8396	21	308	321	LVTWYDNEFGYSNR	83.05000305	100
	1763.8024	1763.8396	21	308	321	LVTWYDNEFGYSNR		

	Protein Name	Species	Accession Number	Protein MW	Protein PI	Peptide Count	Protein Score	Protein Score C.I. %
**1016**	Glyceraldehyde-3-phosphate dehydrogenase	*Danio rerio*	G3P_DANRE	35761.30078	8.2	2	70.40000153	99.23802433
	Calculated Mass	Observed Mass	Match Error PPM	Start Sequence	End Sequence	Sequence	Ion Score	Ion Score C.I.

				Position	Position			%
	1495.8479	1495.8795	21	233	246	VPTPNVSVVDLTVR	16.55999947	0
	1495.8479	1495.8795	21	233	246	VPTPNVSVVDLTVR		
	1763.8024	1763.8206	10	308	321	LVTWYDNEFGYSNR	31.29000092	20.464
	1763.8024	1763.8206	10	308	321	LVTWYDNEFGYSNR		

	Protein Name	Species	Accession Number	Protein MW	Protein PI	Peptide Count	Protein Score	Protein Score C.I. %
**1780**	Heat shock 70 kDa protein	*Oncorhynchus tshawytscha*	HSP70_ONCTS	70931.89844	5.42	6	118	100
	Calculated Mass	Observed Mass	Match Error PPM	Start Sequence Position	End Sequence Position	Sequence	Ion Score	Ion Score C.I. %
	1109.5739	1109.5743	0	351	359	LLQDFFNGR	24.79000092	0
	1109.5739	1109.5743	0	351	359	LLQDFFNGR		
	1487.7013	1487.7036	2	39	51	TTPSYVAFTDTER		
	1675.7307	1675.7417	7	223	238	ATAGDTHLGGEDFDNR		
	1880.9501	1880.9432	-4	141	157	VSNAVITVPAYFNDSQR	53.93999863	99.783
	1880.9501	1880.9432	-4	141	157	VSNAVITVPAYFNDSQR		
	2309.1885	2309.1428	-20	141	161	VSNAVITVPAYFNDSQRQATK		

	Protein Name	Species	Accession Number	Protein MW	Protein PI	Peptide Count	Protein Score	Protein Score C.I. %
**1397**	Adenylate kinase isoenzyme 1	*Cyprinus carpio*	KAD1_CYPCA	21475.33984	6.64	2	108	100
	Calculated Mass	Observed Mass	Match Error PPM	Start Sequence Position	End Sequence Position	Sequence	Ion Score	Ion Score C.I. %
	1053.5364	1053.5082	-27	89	97	GYLIDGYPR		
	1481.7383	1481.7144	-16	32	44	YGYTHLSSGDLLR	94.08999634	100
	1481.7383	1481.7144	-16	32	44	YGYTHLSSGDLLR		

	Protein Name	Species	Accession Number	Protein MW	Protein PI	Peptide Count	Protein Score	Protein Score C.I. %
**1783**	Glucose-6-phosphate isomerase	*Bos taurus*	G6PI_BOVIN	62815.03906	7.33	5	75.80000305	99.78024389
	Calculated Mass	Observed Mass	Match Error PPM	Start Sequence Position	End Sequence Position	Sequence	Ion Score	Ion Score C.I. %
	1424.7029	1424.708	4	29	39	LFEGDRDRFNR		
	1586.8325	1586.8656	21	181	194	VWFVSNIDGTHIAK	66.27999878	99.997
	1586.8325	1586.8656	21	181	194	VWFVSNIDGTHIAK		
	1618.7609	1618.7562	-3	15	27	TWYEQHGSDLNLR		
	1655.8123	1655.751	-37	212	226	TFTTQETITNAETAK		
	1954.0062	1953.9714	-18	107	124	SNAPILVDGKDVMPEVNR		

	Protein Name	Species	Accession Number	Protein MW	Protein PI	Peptide Count	Protein Score	Protein Score C.I. %
**672**	Glutamate dehydrogenase, mitochondrial	*Chaenocephalus aceratus*	DHE3_CHAAC	55359.08984	7.34	2	84.40000153	99.9696652
	Calculated Mass	Observed Mass	Match Error PPM	Start Sequence Position	End Sequence Position	Sequence	Ion Score	Ion Score C.I. %
	1059.5331	1059.5177	-15	391	399	NLNHVSYGR		
	1652.8755	1652.8666	-5	427	442	QGGPIPVVPTADFQAR	68.02999878	99.989
	1652.8755	1652.8666	-5	427	442	QGGPIPVVPTADFQAR		

Table S4. Average normalized spot volumes extracted from Same Spots concerning the proteomic analysis carried out in the muscle of *Sparus aurata* exposed to 18ºC, 24ºC and 30ºC for 21 days. Bold lines indicate spots identified through mass spectrometry.

Spot	Identification	Anova (p)	Fold	Average Normalised Volumes		
				18ºC	24ºC	30ºC
**1781**	**Heat shock 70kDa protein**	**4,928e-006**	**2,5**	**2,031e+006**	**2,043e+006**	**5,054e+006**
**1780**	**Heat shock 70 kDa protein**	**6,131e-005**	**2,2**	**3,516e+006**	**3,236e+006**	**7,058e+006**
**475**	**Heat shock cognate 71 kDa protein**	**0,001**	**1,6**	**4,290e+006**	**4,321e+006**	**6,701e+006**
1836		0,001	1,5	1,359e+006	1,216e+006	1,826e+006
1792		0,002	2,1	2,656e+006	1,759e+006	1,243e+006
**1086**	**Sterile alpha motif domain-containing protein 9-like**	**0,002**	**1,3**	**1,866e+006**	**1,479e+006**	**1,982e+006**
1246		0,002	3,3	4,404e+006	7,147e+006	1,437e+007
**1389**	**Adenylate kinase isoenzyme 1**	**0,002**	**1,8**	**2,476e+006**	**2,459e+006**	**4,337e+006**
627		0,003	1,6	4,882e+006	7,831e+006	7,149e+006
472		0,004	1,5	1,561e+006	1,721e+006	2,278e+006
1448		0,005	1,5	2,836e+006	2,378e+006	3,526e+006
**999**	**Glyceraldehyde-3-phosphate dehydrogenase**	**0,006**	**2,1**	**4,654e+006**	**5,416e+006**	**9,946e+006**
575		0,006	1,6	2,388e+006	1,703e+006	1,507e+006
641		0,006	1,6	1,350e+006	9,300e+005	1,475e+006
**1007**	**Glyceraldehyde-3-phosphate dehydrogenase**	**0,006**	**1,9**	**5,109e+006**	**6,707e+006**	**9,902e+006**
**1783**	**Glucose-6-phosphate isomerase**	**0,008**	**1,3**	**2,235e+007**	**1,810e+007**	**2,371e+007**
1080		0,008	1,4	1,670e+006	1,215e+006	1,152e+006
792		0,009	1,7	4,025e+005	2,336e+005	2,728e+005
990		0,009	2,0	1,635e+007	1,476e+007	2,988e+007
1859		0,009	1,6	3,945e+005	6,440e+005	5,598e+005
589		0,010	1,7	2,141e+006	1,777e+006	3,058e+006
326		0,010	2,1	6,542e+005	3,576e+005	3,147e+005
**989**	**Glyceraldehyde-3-phosphate dehydrogenase (Fragment)**	**0,010**	**1,6**	**3,466e+006**	**3,185e+006**	**5,145e+006**
1356		0,019	1,4	1,461e+006	1,019e+006	1,222e+006
1236		0,019	1,3	1,531e+006	1,210e+006	1,513e+006
1341		0,021	1,5	3,396e+005	3,266e+005	4,814e+005
642		0,021	2,4	1,036e+006	5,574e+005	4,344e+005

Spot	Identification	Anova (p)	Fold	Average Normalised Volumes		
				18°C	24°C	30°C
434		0,025	1,4	9,685e+005	1,368e+006	1,044e+006
1860		0,025	1,3	1,600e+006	1,884e+006	1,460e+006
**1017**	**Glyceraldehyde-3-phosphate dehydrogenase**	**0,025**	**1,6**	**6,087e+006**	**8,252e+006**	**9,962e+006**
**672**	**Glutamate dehydrogenase, mitochondrial**	**0,027**	**1,9**	**1,299e+006**	**9,467e+005**	**6,963e+005**
**1016**	**Glyceraldehyde-3-phosphate dehydrogenase**	**0,030**	**1,6**	**7,847e+006**	**1,048e+007**	**1,260e+007**
645		0,031	2,2	6,132e+005	3,914e+005	2,781e+005
**1331**	**Triosephosphate isomerase (Fragments)**	**0,032**	**2,0**	**1,500e+007**	**1,020e+007**	**1,996e+007**
619		0,033	1,4	1,210e+006	1,315e+006	1,649e+006
**1397**	**Adenylate kinase isoenzyme 1**	**0,034**	**1,7**	**2,612e+006**	**1,513e+006**	**2,156e+006**
950		0,039	2,0	5,009e+005	4,562e+005	9,268e+005
1192		0,039	1,5	1,772e+006	1,213e+006	1,783e+006
1209		0,043	1,7	2,281e+005	2,109e+005	3,523e+005
1784		0,044	1,4	2,974e+006	3,428e+006	2,412e+006
592		0,045	1,4	1,412e+006	1,716e+006	2,027e+006
636		0,046	2,0	1,099e+006	6,429e+005	5,587e+005

Fig. S1 a) Experimental setup (not to scale). Re-circulating system (total of 2,000L) with six 70L white polyvinyl tanks (35 × 35 × 55 cm) for juveniles of *Sparus aurata* (n=6 individuals.tank^{-1}). Inflow of clean water in each tank was 300 mL.min^{-1}. All the tanks were filled with clean and aerated sea water (95-100% air saturation), with a constant temperature of 18±0.5°C, 24±0.5°C and 30±0.5°C (n=2 tanks for each temperature). Salinity was kept at 35‰ and pH at 8±0.01. All tanks were provided with a filter (ELITE Underwater Mini-Filter Hagen, 220L.h^{-1}). Note: *S. aurata* drawings by D. Madeira. **b)** Timeline and sampling scheme of the experiment. The fish were euthanized through cervical transection at day 14, 21 and 28 for collection of muscle. At each time point, four individuals were randomly sampled (2 from each tank). T – Timepoint in days.

a)

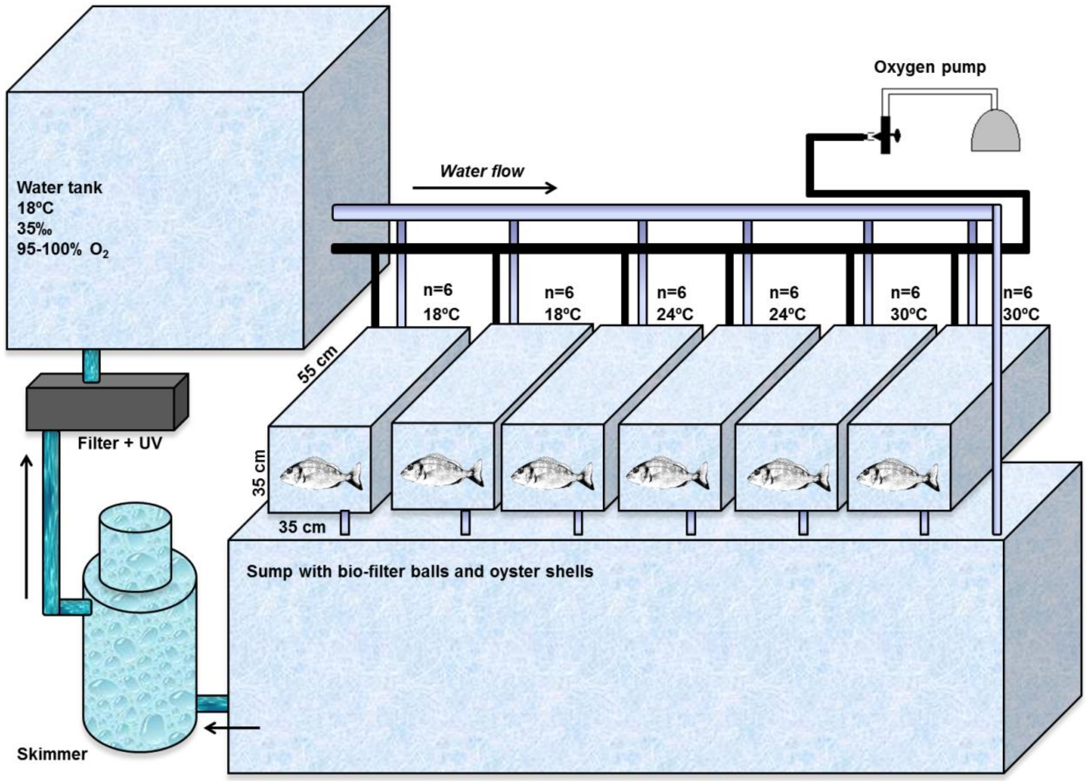

b)

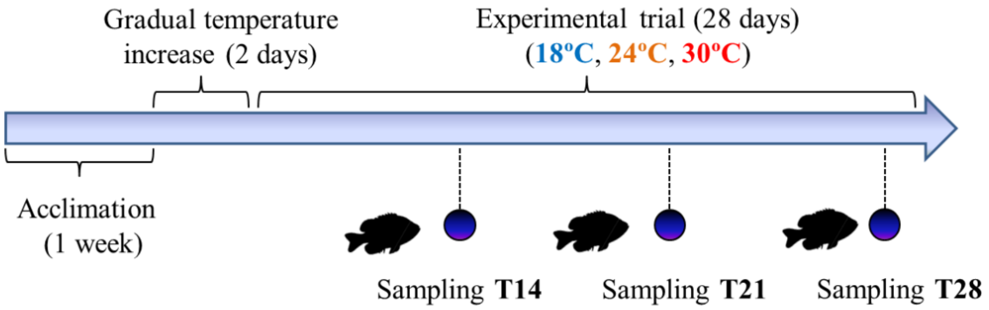
Gradual temperature increase (2 days)
Experimental trial (28 days)
(18ºC, 24ºC, 30ºC)
Acclimation (1 week)
Sampling T14
Sampling T21
Sampling T28

Fig. S2 Comparison between 14 and 21 days of exposure in terms of percent distribution of proteins into functional classes (at 14 days: total of 21 proteins; at 21 days: total of 14 proteins).

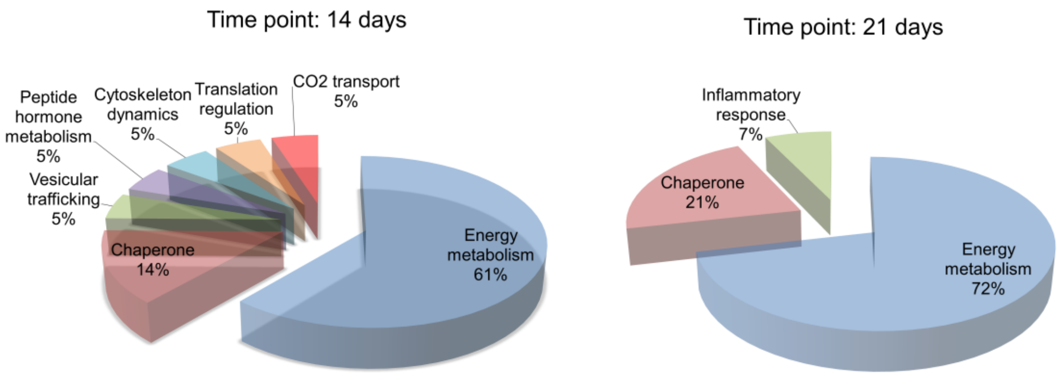

Supplement: Supplementary file 1 [file DataSheet1.pdf]
